# Application of qualitative and quantitative uncertainty assessment tools in developing ranges of plausible toxicity values for 2,3,7,8‐tetrachlorodibenzo‐p‐dioxin

**DOI:** 10.1002/jat.3814

**Published:** 2019-06-30

**Authors:** Daniele Wikoff, Laurie Haws, Caroline Ring, Robert Budinsky

**Affiliations:** ^1^ ToxStrategies, Inc. Asheville North Carolina; ^2^ ToxStrategies, Inc. Austin Texas; ^3^ Dow Chemical Midland Michigan

**Keywords:** reference dose, risk assessment, TCDD, uncertainty

## Abstract

Increasing interest in characterizing risk assessment uncertainty is highlighted by recent recommendations from the National Academy of Sciences. In this paper we demonstrate the utility of applying qualitative and quantitative methods for assessing uncertainty to enhance risk‐based decision‐making for 2,3,7,8‐tetrachlorodibenzo‐p‐dioxin. The approach involved deconstructing the reference dose (RfD) via evaluation of the different assumptions, options, models and methods associated with derivation of the value, culminating in the development of a plausible range of potential values based on such areas of uncertainty. The results demonstrate that overall RfD uncertainty was high based on limitations in the process for selection (e.g., compliance with inclusion criteria related to internal validity of the co‐critical studies, consistency with other studies), external validity (e.g., generalizing findings of acute, high‐dose exposure scenarios to the general population), and selection and classification of the point of departure using data from the individual studies (e.g., lack of statistical and clinical significance). Building on sensitivity analyses conducted by the US Environmental Protection Agency in 2012, the resulting estimates of RfD values that account for the uncertainties ranged from ~1.5 to 179 pg/kg/day. It is anticipated that the range of RfDs presented herein, along with the characterization of uncertainties, will improve risk assessments of dioxins and provide important information to risk managers, because reliance on a single toxicity value limits the information needed for making decisions and gives a false sense of precision and accuracy.

## Abstract 2

Overall uncertainty in the reference dose (RfD) for 2,3,7,8‐tetrachlorodibenzo‐p‐dioxin was found to be high; quantitative evaluation using different assumptions, options, models and methods produced alternative RfD values ranging from ~1.5 to 179 pg/kg/day. The purpose of this assessment was to evaluate the uncertainty in the RfD using qualitative and quantitative approaches. Building on sensitivity analyses conducted by the US Environmental Protection Agency in 2012, resulting estimates of RfD values ranged from ~1.5 to 179 pg/kg/day when accounting for uncertainty in candidate study validity, selection of points of departure, classification of points of departure as no‐effect or lowest‐effect levels, and uncertainty factors. This provides important information to the risk manager and serves to enhance risk‐based decision‐making.

## 1 INTRODUCTION

The Integrated Risk Information System (IRIS) program of the US Environmental Protection Agency (USEPA) supports the agency mission of protecting human health and the environment by identifying and characterizing the health hazards of chemicals found in the environment. One of the key outputs of an IRIS assessment is the development of toxicity values, including reference doses (RfDs) or concentrations, oral cancer slope factors and inhalation unit risk values. These toxicity values are applied in risk assessments and directly impact risk management decisions regarding mitigation of chemical exposures. The IRIS toxicity values are used across agency programs, as well as by other federal, state and local health and environmental agencies, and a host of international health organizations (USEPA, 2018).

In 2014, at the request of Congress, the National Academy of Sciences (NAS) reviewed the IRIS program and offered suggestions for improvements to the IRIS process (National Academies of Science, Engineering, and Medicine, 2014). NAS recommended establishing guidelines for study selection, justifying assumptions and models to determine points of departure (PODs), describing modeling processes, assessing sensitivities in estimates, and characterizing confidence and uncertainty. Their recommendations emphasized the role of evidence evaluation and integration as they relate to the development of toxicity values. In doing so, National Academies of Science, Engineering, and Medicine (2014) stated, “EPA will need to make the best use of the totality of the evidence with increased attention to distinguishing the quality and relevance of studies assessing human dose‐response relationships.” NAS further discussed the potential for multiple values used as PODs, such as a central estimate and a lower bound, because such an approach provides important information on uncertainty.

The utility of these recommendations has been discussed and demonstrated in recent evaluations, including Bayesian approaches for uncertainty factors (UFs), as well as overall approaches for characterizing and communicating uncertainty (Beck et al., 2016; Simon, Zhu, Dourson, & Beck, 2016; Van Landingham, Mundt, Allen, & Gentry, 2016). Notably, the emphasis on characterization of uncertainty in risk assessment is increasing globally, as highlighted by ongoing efforts from the World Health Organization (WHO, 2017) and the European Food Safety Authority (EFSA, 2018a). However, few applications of such uncertainty evaluations, either in IRIS assessments or in the peer‐reviewed literature, are available.

The objective of the present work was to assesses the uncertainty in the RfD using qualitative and quantitative approaches suggested by National Academies of Science, Engineering, and Medicine (2014) and Beck et al. (2016). Building on sensitivity analyses conducted by USEPA (2012), a number of independent analyses were conducted as part of an extended assessment of uncertainty around the RfD. These include consideration of risk of bias in the two candidate datasets, statistical analysis associated with POD selection, application of Bayesian techniques to UFs and a quantitative characterization as part of developing a range of plausible RfDs reflective of underlying uncertainty. It is anticipated that having a range of values like this will provide risk managers with a better understanding of the level of confidence in the underlying dataset and will provide data that can be used qualitatively or quantitatively when making risk assessment and risk management decisions. This evaluation demonstrates the utility of applying qualitative and quantitative methods for assessing uncertainty to enhance risk‐based decision‐making using an approach that is consistent with NAS recommendations.

## 2 BACKGROUND OF US ENVIRONMENTAL PROTECTION AGENCY REFERENCE DOSE FOR TETRACHLORODIBENZO‐P‐DIOXIN

The USEPA is commended on the substantial effort put in over the course of decades to develop the current RfD for 2,3,7,8‐tetrachlorodibenzo‐p‐dioxin (TCDD). Dioxin and dioxin‐like compounds (DLCs) are some of the most extensively researched substances in the field of toxicology. As a result, the evidence base is voluminous, consisting of more than 15 000 publications in PubMed alone. The USEPA first evaluated TCDD in 1985, and over time, has conducted several re‐evaluations (described in USEPA, 2012). As summarized by USEPA (2012), the most recent analysis, which began in 2003, is accompanied by its own complex series of undertakings when the NAS was asked by the USEPA, as well as other federal agencies, to review the agency's draft reassessment. In 2006, NAS issued the findings of their review, identifying three areas that required improvement: (1) justification for approaches to dose‐response modeling; (2) transparency and clarity in the selection of key datasets for analysis; and (3) transparency, thoroughness and clarity in the quantitative uncertainty analysis (National Academies of Science, Engineering, and Medicine, 2006).

Subsequently, the USEPA published a literature database of peer‐reviewed studies on TCDD toxicity; the agency convened a workshop to identify and address issues related to the dose‐response assessment of TCDD and to ensure that the agency's response to the NAS focused on the key issues and reflected the most meaningful science (USEPA, 2009). A draft report was then issued in 2010: *EPA's Reanalysis of Key Issues Related to Dioxin Toxicity and Response to NAS Comments* (USEPA, 2010a, 2010b). This draft, often referred to as the “Reanalysis,” was then subjected to an external peer review by a USEPA Scientific Advisory Board (SAB), which held a series of public meetings before issuing their report in 2011. In this report, the SAB commended the USEPA for their comprehensive and rigorous process and documented their support for many of the decisions made by the agency. One of the most notable of the SAB comments was related to the feasibility of a comprehensive uncertainty analysis. More specifically, the SAB believed that a comprehensive quantitative uncertainty analysis was feasible and should be conducted. While the EPA's *Reanalysis of Key Issues Related to Dioxin Toxicity and Response to NAS Comments*, Volume 1 (USEPA, 2012) included a quantitative uncertainty analysis associated with the development of PODs, uncertainty associated with other aspects of the assessment that affect the RfD were either not addressed or addressed only qualitatively.

In developing the current RfD for TCDD, USEPA (2012) selected two co‐critical studies—Mocarelli et al. (2008) and Baccarelli et al. (2008)—both of which are associated with the Seveso, Italy, accident, which involved exposures to very high concentrations of TCDD during and shortly after an explosion at a trichlorophenol manufacturing plant in July 1976 (Mocarelli, 2001). Mocarelli et al. (2008) reported on altered sperm concentrations and sperm motility in men exposed to TCDD as children during the Seveso accident. Baccarelli et al. (2008) reported on altered thyroid‐stimulating hormone (TSH) levels in newborns from mothers exposed to TCDD during the Seveso accident. From each of these studies, a serum concentration was selected as it related to male reproductive effects or increased TSH in neonates. From these serum concentrations, which are metrics of internal dose (i.e., lipid‐adjusted serum concentrations of TCDD) measured at a single point in time, estimates of daily exposure (administered dose) were derived using a kinetic model. These daily intakes were identified as the PODs, each of which was characterized as the lowest‐observed‐adverse‐effect level (LOAEL). From the POD, UFs were applied to derive a final RfD of 0.7 pg/kg/day (Table 1).

**Table 1:** Co‐critical study information and USEPA's derivation of the RfD values for TCDD

Parameter	Mocarelli et al., (2008)	Baccarelli et al., (2008)
Study type and population	Human, cohort (Seveso, 1976)	Human, cohort (Seveso, 1976)
Endpoint	Serum concentrations associated with decreased sperm concentrations in men exposed as boys aged 1‐9	Maternal serum concentrations associated with increased β‐TSH in neonates
Data selected to characterize exposure‐response	Serum concentration of 68 ppt (median concentration of Q1; measured in 1976) associated with decreased sperm concentrations and decreased sperm motility (measured in 1997‐1998)	Maternal serum concentration of 235 ppt (estimated from regression plot based on measurements from 1992 to 1998 extrapolated to time of birth) associated with neonate β‐TSH >5 μU/mL (1994‐2005)
↓↓ Kinetic model used by USEPA to determine daily intake associated with serum concentrations ↓↓		
POD = Daily intake associated with serum concentrations	0.020 ng/kg/day (mean of peak exposure [0.032 ng/kg/day] and average exposure over 10‐year critical window [0.0080 ng/kg/day])	0.020 ng/kg/day
POD type	LOAEL	LOAEL
UF	30 UF_L_ = 10; UF_H_ = 3	30 UF_L_ = 10; UF_H_ = 3
RfD	0.7 pg/kg/day	

β‐TSH, blood thyroid‐stimulating hormone; LOAEL, lowest‐observed‐adverse‐effect level; POD, points of departure; RfD, reference dose; UF, uncertainty factor; USEPA, US Environmental Protection Agency.

## 3 GENERAL APPROACH FOR CHARACTERIZING UNCERTAINTY IN THE REFERENCE DOSE

The approach for characterizing uncertainty followed recommendations by National Academies of Science, Engineering, and Medicine (2014) for the analysis and communication of uncertainty. The objective of this approach was to provide “a demonstration of variation in the final toxicity value estimates under different assumptions, options, models, and methods” (National Academies of Science, Engineering, and Medicine, 2014). This approach allows for visualization of uncertainty in each stage of the process, recognizing that those in earlier stages cascade and propagate to later stages. Further guidance on specific approaches to characterizing uncertainty was informed by Beck et al. (2016); these approaches suggest deconstruction of the toxicity value to enhance transparency, presenting PODs and toxicity values in the context of alternatives and evaluating uncertainty in individual elements of the hazard characterization. Recommendations also included developing visual aids and transparent narratives related to confidence and uncertainty.

The areas of uncertainty evaluated herein paralleled the key decisions made in developing the USEPA RfD for TCDD. These areas include:

1. Candidate study selection and evaluation of Mocarelli et al. (2008) and Baccarelli et al. (2008) as co‐critical studies from the overall body of evidence.
2. Selection of the serum concentrations to derive the POD, and classification of the serum concentrations as the no‐observed‐adverse‐effect levels (NOAELs)/LOAELs.
3. Application of a kinetic model to derive the POD from the serum concentrations in the studies.

The uncertainty in the TCDD RfD was characterized both qualitatively and quantitatively by deconstructing and identifying uncertainties, and then reconstructing potential RfD values using different assumptions, options, models and methods that captured the key areas of uncertainty (Beck et al., 2016; National Academies of Science, Engineering, and Medicine, 2014). For the qualitative assessment, uncertainties in the selection of candidate studies, as well as the characterization and use of the data from the co‐critical studies, are considered. To do so, the studies are discussed relative to the inclusion criteria implemented by USEPA (2012), which involved critical appraisal of the study quality and the overall body of evidence for each given endpoint (e.g., consistency). Notably, uncertainty as it related to study quality involved assessment of external validity (the degree to which the results of a study can be generalized to groups other than those in the given study) and internal validity (often measured by risk of bias, an evaluation of the potential for a systematic error or deviation from true effect). Herein, the Risk of Bias tool for the National Toxicology Program (Office of Health Assessment and Translation, 2015a, 2015b) was used to guide the characterization of uncertainty associated with internal validity.

Continuing with the deconstruction of the toxicity values, the uncertainty in the selection of PODs (or serum concentrations used to develop the PODs) from the co‐critical studies was characterized qualitatively, and the PODs were classified as LOAEL or NOAEL values. When possible, alternative classifications or alternative values were considered quantitatively based on aspects such as statistical significance, clinical significance and level of severity (e.g., determination as to whether a response is adverse). For the kinetic modeling, assumptions and output were considered in the context of established methods and integration of the concentration‐ and age‐dependent kinetics for TCDD.

Potential ranges of RfD values were then determined using multiple approaches. First, alternative RfD values were calculated using a combination of deterministic calculations similar to the “sensitivity tree” approach used by USEPA (2012), in which uncertainties in POD or characterization of the POD as a NOAEL or LOAEL were explored. Then, as per NAS recommendations (National Academies of Science, Engineering, and Medicine, 2014), Bayesian approaches were applied to characterize the distribution of RfD values, which were subsequently compared with the current RfD. A recently published approach for developing probabilistic dose‐response characterizations based on animal data (Chiu et al., 2018) was considered but not implemented as the data examined here are from human studies.

It should be noted that our assessment focused on uncertainty characterization specific to the RfD and the two co‐critical studies rather than providing a full weight‐of‐evidence assessment related to hazards and risks associated with TCDD. Therefore, much of the information conveyed in the USEPA Dioxin Reassessment is not reflected here.

## 4 UNCERTAINTIES RELATED TO SPERM ENDPOINT (MOCARELLI ET AL., 2008)

### 4.1 Mocarelli et al. (2008) background

Mocarelli et al. (2008) investigated semen quality parameters and reproductive hormone levels in adult males several decades after their exposure to TCDD as children during the Seveso accident in 1976. The authors reported that males aged 1‐9 years at the time of exposure were reported to have reductions in sperm concentration, percentage progressive motility and total motile sperm count as adults. In contrast, males aged 10‐17 years at the time of exposure were reported to have *increased* sperm counts and total motile sperm. The authors concluded that the reductions in sperm count and motility in males aged 1‐9 years at the time of exposure were permanent and occurred at serum TCDD concentrations <68 ppt (median TCDD concentration of the first quartile of men aged 1‐9 years during the Seveso incident). The USEPA selected the serum concentration of 68 ppt as the concentration on which to base the POD; this value was characterized as a LOAEL for decreased sperm concentrations and decreased sperm motility. Using a kinetic model, a daily intake of 0.020 ng/kg/day (mean of peak and average exposure over a 10‐year critical window) was derived. This POD was divided by a composite UF of 30 to derive the candidate RfD of 0.7 pg/kg/day (Table 1). Uncertainties associated with study selection and evaluation, POD selection and classification, and kinetic modeling are summarized in Table 2 and discussed below.

**Table 2:** Inclusion criteria imposed by USEPA (2012) and Mocarelli et al.'s (2008) compliance with the inclusion criteria

Inclusion criteria (USEPA, 2012)	Mocarelli et al.'s (2008) compliance with the inclusion criteria
Study is published in the peer‐reviewed scientific literature and provides an appropriate discussion of data collection and analysis methods, as well as sufficient detail to allow consideration of its strengths and limitations.	Significant exclusion bias limits ability to consider approach and findings fully; 10 men with serum concentrations of >2000 ppt, median concentration of 6350 ppt were excluded from the analyses without explanation of potential impact (authors did not provide any information regarding sperm concentrations; it is anticipated that this would have altered the median serum concentrations of the quartile data used in development of a candidate RfD).
Exposure is primarily to TCDD, rather than DLCs, and can be quantified so that dose‐response relationships can be assessed for non‐fatal adverse Because all epidemiologic cohorts have background exposures to DLCs, in which TCDD is a minor component, only those studies for which TCDD exposure is well above background will qualify for dose‐response modeling. To the extent to which background DLC exposure becomes more significant with respect to TCDD exposure, limited quantitative assessment of DLC background exposures may be necessary endpoints.	Exposures to other DLCs were significant: USEPA estimated that equivalent POD based on TEQ was ~140 ppt (vs. TCDD‐only POD of 68 ppt); thus, ~52% of the total TEQs were DLCs other than TCDD
Effective dose and oral exposure must be quantifiable. Timing of the measurement of health endpoints (i.e., the response) also must be consistent with current biological understanding of the endpoint and its progression.	Exposure to TCDD based on accidental explosion, which involved a bolus inhalation exposure in addition to oral and dermal exposure. Exposure dose utilized by USEPA did not reflect the peak exposure. Results not biologically consistent; opposite responses observed in males aged 1‐9 vs. 10‐17 years.
Methods used to ascertain health outcomes are clearly identified and unbiased (e.g., outcome classification was made―blinded to exposure levels of the study participants).	Well‐documented that a single sperm sample clinically insufficient to characterize sperm quality Unclear whether all endpoints assessed via blinded procedures
Risk estimates generated from the study are not susceptible to important biases arising from an inability to control or account for confounding factors or other sources of bias (e.g., selection or information bias) arising from limitations of the study design, data collection or statistical analysis.	Potential selection bias in control group; no information provided on geographic origins or ethnicity (unclear whether recruited from same eligible population)
Study demonstrated an association between TCDD and an adverse health endpoint (assuming minimal misclassification of exposure and absence of important biases) with some suggestion of an exposure‐response relationship.	No statistical tests were conducted by the authors for the data used to develop the candidate RfD (quartile analyses) Single sample of sperm concentration not a measure of adversity; all sperm concentrations were within clinically acceptable levels related to fertility
Exposure assessment method is clearly described and can be expected to provide adequate characterization of exposure, with assignment of individual‐level exposures within a study (e.g., based on biomarker data or based on a job‐exposure‐matrix approach). Limitations and uncertainties in the exposure assessment are considered.	Exposure to TCDD was not measured in control group (concentrations were assumed <15 ppt in 1976)
Size and follow‐up period of a cohort study are large enough and long enough, respectively, to yield sufficiently precise estimates for use in development of quantitative risk estimates and to ensure adequate statistical power to limit the possibility of not detecting an association that might be present. Similar considerations regarding sample size and statistical precision and power apply to other study designs such as case‐control studies.	Independent analysis of the sample size and sampling design demonstrates a lack of statistical power to support conclusions.

DLC, dioxin‐like compound; POD, point of departure; RfD, reference dose; TCDD, tetrachlorodibenzo‐p‐dioxin; TEQ, toxic equivalency; USEPA, US Environmental Protection Agency.

#### 4.1.1 Uncertainty related to selection of Mocarelli et al. (2008) as a candidate study

The selection of the co‐critical studies is not consistent with the systematic review process more recently proposed by National Academies of Science, Engineering, and Medicine (2014). Since the time of the release of the RfD in 2012, the NAS has described a systematic review as an approach to identify, evaluate and integrate the evidence—steps that support hazard identification and dose‐response assessment. The earlier approach implemented by USEPA used a systematic search (but not a systematic review). That is, the systematic search was conducted using specific inclusion criteria (Table 2) related to identifying studies that characterize the dose‐response relationship for TCDD. Thus, the study was selected in the absence of context with regard to how it related to other studies characterizing the same endpoint. The process did not involve an overall characterization of potential hazard, and subsequent selection of a candidate study to represent the hazard.

Many of the inclusion criteria (Table 2) involve aspects of internal validity of a study. Internal validity, often evaluated by risk of bias (OHAT, 2015), is a significant component of the critical appraisal process recommended by National Academies of Science, Engineering, and Medicine (2014). It provides a measure of whether the design and conduct of a study compromised the credibility of the link between exposure and outcome. Notably, National Academies of Science, Engineering, and Medicine (2014) is recommending that a risk‐of‐bias assessment be conducted on studies that are used by USEPA as primary data sources for hazard identification and dose‐response assessment. Thus, the use of some aspects of internal validity as means of inclusion criteria would contribute to increased confidence and quality in an assessment. When these more recent criteria implemented for inclusion were appraised for Mocarelli et al. (2008), significant uncertainties were identified (Table 2).

The sperm quality data reported in Mocarelli et al. (2008) are subject to uncontrolled sources of methodological error (e.g., high risk of bias) and, thus, were found to have substantial uncertainty related to the inclusion criteria. Specifically, the outcome (sperm quality) was not evaluated using a valid measure; all the sperm quality data are based on a single sample from each subject. This is contrary to guidance from the WHO (2010, p. 8), which states, “It is impossible to characterize a man's semen quality from evaluation of a single semen sample.” To account for intrasubject variation in sperm concentrations, multiple sequential samples are needed over time. More specifically, WHO (2010, p. 8) states, “It is helpful to examine two or three samples to obtain baseline data.” Thus, a single sperm sample is not valid for statistical comparisons between groups. The USEPA acknowledged this limitation in the measurement of the outcome, stating that “… the collection of a single semen sample is not suitable for accurate evaluation of semen effects in an individual …” (USEPA, 2012, p. C‐124) but nonetheless relied on Mocarelli et al. (2008) as a co‐critical study. Further limiting the reliability of the sperm quality data, Mocarelli et al. (2008) indicate that data on the length of abstinence between ejaculations were obtained, but the authors do not fully report these data. Abstinence is a major determinant of the variation in seminal parameters, and understanding the potential impact of differences in abstinence length is important to understanding the results (Carlsen, Petersen, Andersson, & Skakkebaek, 2004; WHO, 2010).

With respect to exposure bias, serum concentrations of TCDD were not measured for the control group (but were measured for the exposed individuals). Mocarelli et al. (2008) assumed that the serum TCDD concentrations for the comparison groups were ≤15 ppt in 1976‐1977. EFSA (2018b) has also recently cited the poor exposure information for the control group as a potential source of bias. Furthermore, with respect to the comparison (or control) group, no information was provided regarding the geographic origins or ethnicity, and, thus, it is unclear whether participants were recruited from the same eligible population (i.e., bias related to selection of control groups). These elements are known to influence the validity of the outcome measurements (sperm counts vary by geographic region) (Fisch, Ikeguchi, & Goluboff, 1996; Safe, 2000), as do exposure measurements (dioxin concentrations vary by ethnicity), respectively (Bichteler, Wikoff, Loko, & Harris, 2017). EFSA (2018b) also recently cited the lack of information on the likeness of the control group as a probable source of bias in this study.

Furthermore, there is direct evidence of exclusion bias; the original study authors excluded men that were characterized as “very highly exposed” (Mocarelli et al., 2008, p. 71). Specifically, 10 men aged 1‐9 years old in 1976 were excluded from the analysis; this group had a median serum concentration of 6350 ppt (a concentration one to two orders of magnitude greater than the medians of the first [68] and fourth quartiles [733], respectively). The authors did not provide a rationale for excluding these participants; no information was provided regarding the sperm quality or the impact of excluding these participants on the exposure‐response relationship. EFSA (2018b) has also recently cited this as a potential bias in the study methods. In addition to attrition/exclusion bias, there probably was a high risk of selection bias. The overall participation rate of 33% is low and is different from the participation rate of the control groups (49%). Neither Mocarelli et al. (2008) nor USEPA (2012) addressed these factors as part of an uncertainty analysis. Notably, EFSA also recently described a probably high risk of bias related to the selection domain due to these issues with participation rate (EFSA, 2018b; Annex A.9.1).

There is also a high level of uncertainty related to the generalizability of the exposures and effects observed in Mocarelli et al. (2008) to represent the general population exposure‐response. Specifically, there is uncertainty regarding reliance on a study involving high‐dose, acute exposure from what is described as a toxic cloud following the Seveso incident (Pesatori, 1995; Pesatori et al., 2003; Signorini et al., 2000). The experimental animal literature demonstrates the role of dose in the relationship between high‐dose, bolus exposure (vs. chronic low‐dose exposures) and male reproductive effects (Foster, Maharaj‐Briceno, & Cyr, 2010). In a comprehensive review of studies examining the effect of in utero and developmental exposure to TCDD on male rat reproductive system parameters (Bell et al., 2010), the authors state that acute exposures lead to higher hepatic sequestration and less fetal distribution. This supports uncertainty in translating exposures from Seveso (e.g., acute exposure interval before evacuations), to predicting human responses where exposure occurs chronically, and to low levels, via dietary intake.

#### 4.1.2 Uncertainty in the point of departure from Mocarelli et al. (2008) based on lack of statistical significance

The sperm concentrations of exposed men evaluated by quartile were not subjected to a statistical evaluation by the study authors or by the USEPA. The USEPA attempted to obtain the original data from the Mocarelli et al. (2008), noting that the first author was also a member of the agency's SAB charged with reviewing the USEPA assessment (USEPA, 2010c). However, the study authors did not make these data available. As such, no tests could be conducted to determine whether the median serum concentration of Q1—the data point used to develop the candidate RfD—was statistically different from the reference group. Because the data were presented only in graphical format, the USEPA *estimated* the decrease in sperm concentrations ~25% (Q1), ~25% (Q2), ~21% (Q3) and ~33% (Q4) relative to the control group—a trend that is not indicative of a dose‐response. Furthermore, when describing the data, the USEPA described the sperm concentration changes in the second, third and fourth quartiles as “minimal” relative to the first quartile (USEPA, 2012, pp. 4‐58), despite significant increases in TCDD serum concentrations. It is notable that in their recent review, EFSA (2018b) also specifically indicated a lack of dose‐response relationship reported in this study.

**Figure 1:**
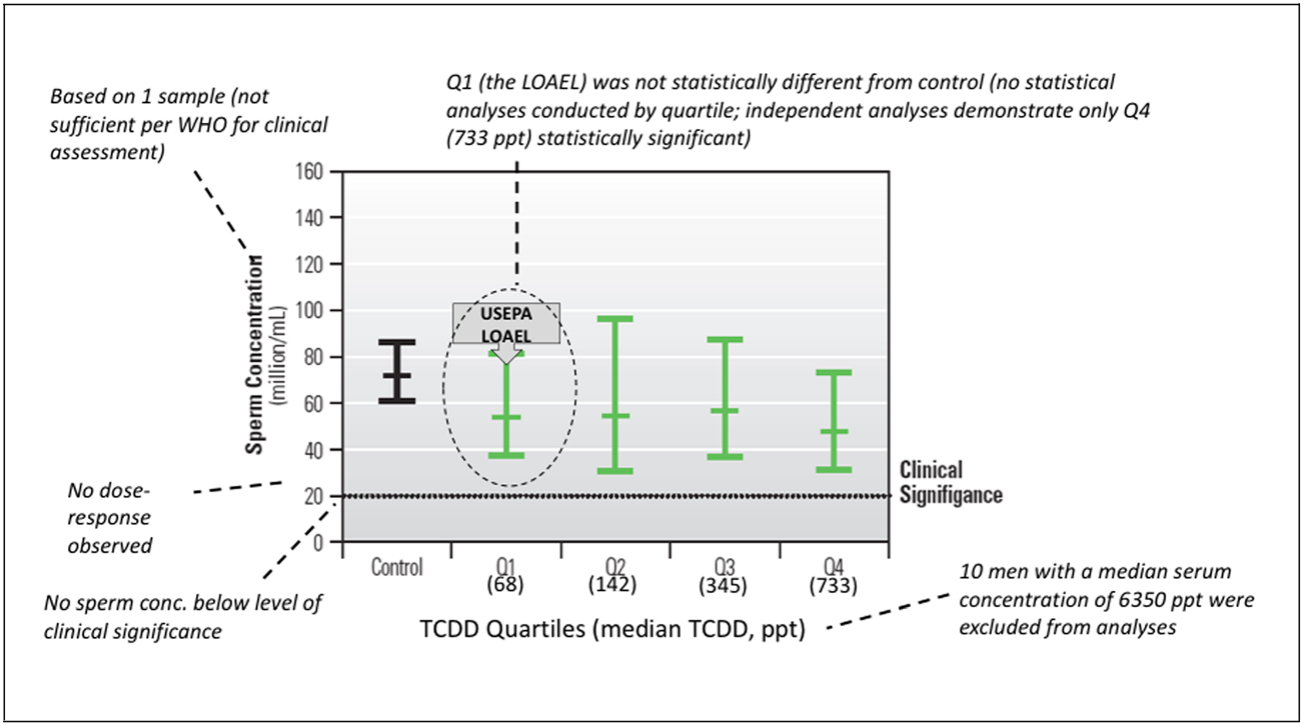
Characterization of key uncertainties in sperm data. Adapted from Figure 3A in Mocarelli et al. (2008); sperm concentration (adjusted mean and 95% confidence interval) for exposed men 1‐9 years old in 1976 and sampled for semen quality indices in 1998. LOAEL, lowest‐observed‐adverse‐effect level; TCDD, 2,3,7,8‐tetrachlorodibenzo‐p‐dioxin; USEPA, US Environmental Protection Agency [Colour figure can be viewed at wileyonlinelibrary.com]

In our assessment, it was determined that the first quartile group was not statistically significantly different from the control group for either measure (Data S1; see Supporting Information). The only quartile to achieve statistical significance was Q4; the median serum concentration in Q4 was 733 ppt (Figure 1). Thus, if using classical designations based on statistical significance, the LOAEL from Mocarelli et al. (2008) would be 733 ppt (median of Q4), and the NOAEL would be 345 ppt (median of Q3). The impact of using a statistically significant finding would result in a ~10‐fold higher serum level based on a LOAEL (and the UF = 10 for UF_L_ retained) or, alternatively, a fivefold higher serum level based on a NOAEL coupled with a 10‐fold lower composite UF (UF_L_ would not be needed). *At a minimum*, the evaluation of statistical significance would suggest that the serum concentration utilized in the development of the RfD (68 ppt) is a NOAEL instead of a LOAEL, as characterized by the USEPA, because it is not statistically different from the control. The classification of 68 ppt as a NOAEL vs. LOAEL, alone, has a significant impact on the RfD—removal of the 10× UF for UF_L_ results in an RfD of 7 pg/kg/day (vs. 0.7).

Statistical significance for the cohort (vs. by quartile) can also be considered. Mocarelli et al. (2008) report an unadjusted sperm concentration mean of 53.6 million/mL (mean ± SD, 21.8‐131.8 million/mL) across all exposed men from the cohort aged 22‐31 years (i.e., those exposed when aged 1‐9 years; regardless of TCDD concentration/assignment by quartile). In the control group, the authors reported a mean concentration of 72.5 million/mL (mean ± SD, 31.7‐165.9 million/mL) in men of similar age but unknown geographic region. The comparison of sperm concentrations for all exposed men aged 22‐31 years (“exposed group” regardless of the exposure level) was reported statistically significant (*P* = 0.025). Thus, the statistically significant finding applies to the evaluation of the entire exposed group (median 210 ppt); the same finding was not reported for a comparison limited to the first quartile (Q1) of the exposed group (median 68 ppt).

#### 4.1.3 Uncertainty in the points of departure based on lack of clinical significance (i.e., lack of adversity) of the sperm deficits in Mocarelli et al. (2008)

All of the sperm concentrations reported by Mocarelli et al. (2008) were within the normal range of variability and are above concentrations considered clinically significant and associated with reduced fertility. As such, the sperm concentrations reported by Mocarelli et al. (2008) should not be considered adverse. All of the mean values shown in Figure 1 are well above 20 million/mL, which is the level identified by USEPA (2012) as the guideline used by WHO (1980) for determining clinically significant deficits in sperm concentrations (i.e., levels <20 million/mL are associated with increased male infertility). However, USEPA (2012) did not discuss more recent guidance by WHO (2010), in which a lower limit of 15 million sperm/mL is cited as a reference value for clinically significant deficits in sperm concentration (with a range of 12‐16 million/mL; WHO, 2010). Regardless, none of the mean sperm concentrations reported by Mocarelli et al. (2008) was lower than any of these reference values, not even in Q4, which was the only quartile determined statistically different from controls in this current assessment (Figure 1). Furthermore, all of the mean values shown in Figure 1 are, in fact, >40 million/mL, which suggests that the entire cohort of exposed men have sperm concentrations above the 25th percentile of the WHO (2010) reference range for men whose partners became pregnant within 20 months of discontinuing contraceptive use. This uncertainty in the adversity of the observed decreases in sperm concentration was acknowledged by the USEPA, which stated, “A decrease in sperm concentration of 25% likely would not have clinical significance for a typical individual …” (USEPA, 2012, pp. 4‐59).

#### 4.1.4 Uncertainty in the points of departure based on shortcomings in the characterization of exposure

The measures of exposure in Mocarelli et al. (2008) were blood samples. These samples were drawn months after the acute exposure in July 1976; because TCDD kinetics are concentration and age dependent, it is likely that the measured levels underpredict exposure at the time of the acute exposure incident. The POD for the EPA reflects an average of the estimated peak dose and the daily dose over a time to achieve the 68‐ppt value. There is uncertainty in using this approach to characterize the exposure metric, as any potential effect is probably associated with peak concentrations associated with the acute, high‐dose exposure to dioxins. Such a relationship has been observed in experimental animal data (Foster et al., 2010). As a result, the POD likely reflects an underestimate (i.e., the POD would be higher if these kinetic aspects are considered).

#### 4.1.5 Uncertainty in the points of departure based on lack of dose‐response

Acknowledging that the decrease in sperm concentration in the first quartile of men aged 1‐9 years at time of exposure was not clinically relevant, the USEPA judged the impact on sperm concentration and quality to be biologically significant based on the potential for functional impairment in a population. This is somewhat incongruent with the agency's acknowledgment that there was no clear adverse‐effect level indicating male fertility problems for either of the sperm effects reported in Mocarelli et al. (2008) and that no effects on the reduction in total sperm count or testosterone levels were observed in concert with the altered sperm concentration and motility counts (USEPA, 2012). Nonetheless, the USEPA relied on a rationale that decreases associated with TCDD could lead to shifts in the distributions of such measures in the general population. The SD below the mean for sperm concentrations across the cohort (not for Q1), 21.8 million/mL, was described as falling near the low end of the range of reduced fertility. Thus, the rationale for the effect was supported by data for the entire cohort, rather than for Q1. Subsequently, a more appropriate POD would be based on the serum concentration associated with statistical significance in the cohort (i.e., 210 ppt) vs. that from Q1. This factor alone introduces a threefold higher serum level to calculate the POD.

### 4.2 Uncertainties related to the thyroid‐stimulating hormone endpoint (Baccarelli et al., 2008)

#### 4.2.1 Baccarelli et al. (2008) background

**Figure 2:**
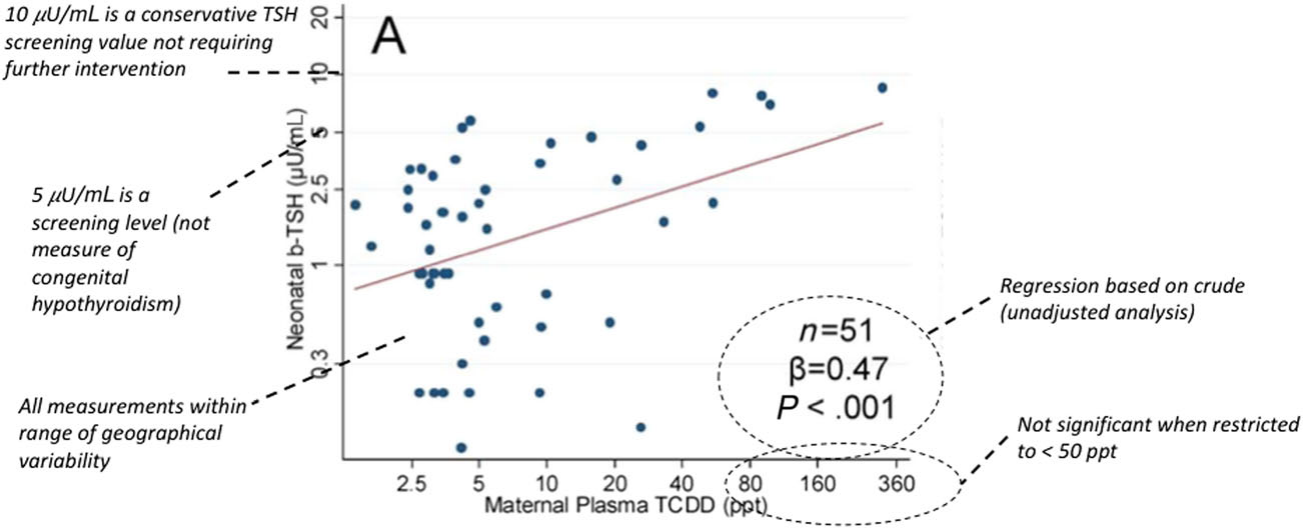
Characterization of key uncertainties in TSH data. Adapted from Figure 2a, Baccarelli et al. (2008); maternal plasma dioxin levels and neonatal β‐TSH. β‐TSH, blood thyroid‐stimulating hormone; TCDD, 2,3,7,8‐tetrachlorodibenzo‐p‐dioxin [Colour figure can be viewed at wileyonlinelibrary.com]

Baccarelli et al. (2008) investigated neonatal blood thyroid‐stimulating hormone (β‐TSH) levels in neonates born to mothers from Seveso. The authors reported that neonatal β‐TSH levels were modified by maternal dioxin exposures. Correlations were reported between neonatal TSH values with both maternal TCDD and toxic equivalency (TEQ). The USEPA relied on information presented in a graphic to characterize the regression of estimated maternal serum concentrations and infant β‐TSH at birth, to determine the critical effect and resulting POD. Using a threshold of 5 μU/mL β‐TSH as an indicator for adverse effects, the USEPA selected the maternal serum concentration of 235 ppt to develop the POD. Using kinetic modeling, a serum concentration of 235 ppt, corresponding to an average daily intake of 0.02 ng/kg/day, was derived. This POD was then divided by a composite UF of 30 to derive the candidate RfD of 0.7 pg/kg/day (Figure 2). Uncertainties associated with study selection and evaluation, POD selection and classification, and kinetic modeling and discussed below.

#### 4.2.2 Uncertainty related to selection of Baccarelli et al. (2008) as a candidate study

As discussed previously, the process implemented by the USEPA in selecting candidate datasets was focused on studies that could be used to characterize the dose‐response relationship for TCDD (vs. hazard characterization). Thus, selection was biased to favor studies with positive findings, and characterization of the body of evidence regarding the relationship between the exposure and increased TSH in humans was not examined, i.e., no systematic characterization of hazard was provided for this endpoint, and no description of consistency with other studies was provided. In this context, the USEPA received a number of peer‐review comments related to the selection of Baccarelli et al. (2008) as a co‐critical study, because it was not consistent with other literature characterizing potential associations between dioxin exposure and thyroid function in neonates, infants and children.

A relatively recent weight‐of‐evidence analysis of human exposures to DLCs and associations with thyroid hormone levels during early development highlight the limitations of the Baccarelli et al. (2008) study (Goodman, Kerper, Boyce, Prueitt, & Rhomberg, 2010). From the 19 studies that specifically evaluated TSH, Goodman et al. (2010) reported a lack of evidence for clear and consistent effects of dioxins on TSH in infants and children, noting that the results were inconsistent, and, in most cases, group differences in TSH were not statistically significant. Among the studies that evaluated TSH from birth to 3 days old, only Baccarelli et al. (2008) reported a significant association with serum TCDD. When interpreting the inconsistencies in findings, Goodman et al. (2010, p. 95) noted that “the range of TSH levels in infants’ blood at 3 days observed in this study was not elevated relative to levels in other studies with lower exposures to dioxins and DLCs, raising questions regarding the potential impact of TCDD on thyroid hormone levels.”

Studies in laboratory animals support the hypothesis of a threshold effect level for TCDD exposure, i.e., TSH (as an indicator of thyroid function) is more consistently altered at high doses, but not at lower, environmentally relevant doses (Goodman et al., 2010; Greene, Hays, & Paustenbach, 2003; Seo et al., 1995; Sewall et al., 1995). The potential role of high‐dose exposure is emphasized by findings from Baccarelli et al. (2008, p. 1136), in which “all positive associations were dependent on the presence in the analyses of participants with very high plasma TCDD level (TCDD >50 ppt, n=5).” When the analyses were restricted to individuals with TCDD levels <50 ppt (i.e., general population exposures), none of the correlations (including TSH) were significant.

Taken together, the inconsistency of the Baccarelli et al. (2008) data relative to data obtained from cohorts other than Seveso, as well as the clear need for high‐dose exposures to obtain a response, highlight the uncertainty in the selection of Baccarelli et al. (2008) as a candidate study. Furthermore, it is plausible that the inconsistency can be explained by the lack of external validity, i.e., the lack of generalizability in using a study involving high‐dose, acute exposure following the Seveso incident to represent dietary exposures in the general population.

With respect to internal validity as it relates to inclusion criteria applied by USEPA (2010), there is likely a high risk of bias in the evaluation and control for confounding. As acknowledged by USEPA (2010a, 2010b), consideration of the confounding effect of maternal iodine intake was not included in the assessment (because it was not available). Although Baccarelli et al. (2008) reported that there was no indication that exposed and unexposed women had differences in uptake, this does not discount the potential confounding of such differences within each group—two different aspects of confounding (OHAT, 2015a, 2015b). Furthermore, the Lombardy region—where Seveso is located—is known to have a borderline‐mild iodide deficiency (5.4%‐6.3%) relative to iodine‐replete populations (<3%) (Corbetta et al., 2009). Given the evidence of regional iodine deficiency (i.e., direct evidence of confounding) and the direct relationship between iodine status and TSH (WHO, 1994) (discussed further below), this is a critical variable in an evaluation of TSH as an outcome, which likely contributes to uncertainty in the selection of Baccarelli et al. (2008) as providing reliable characterizations between TCDD and TSH.

Regarding the evaluation of exposure, the USEPA determined that the uncertainty was too great to utilize neonatal TCDD serum concentrations, instead using maternal serum concentrations. However, the maternal serum concentrations were based on measurements collected between December 1992 and September 1998—several years after the measurements of outcome (collected at birth, January 1994‐June 2005). Thus, instead of using maternal serum concentrations measured at the same time as the outcome, exposure was based on maternal serum concentrations that were extrapolated to the date of delivery by Baccarelli et al. (2008). The combined indirect approach that involves both (1) lack of exposure measurement in the individuals (infants), and (2) consideration that exposure and outcome were not measured at the same time, lend uncertainty in the assessment of exposure.

#### 4.2.3 Uncertainty in the points of departure based on shortcoming in selection and characterization of a serum concentration

### 4.3 Points of departure based solely on extrapolated estimate of unadjusted data

As noted above, the USEPA did not use a value reported by the study authors, but rather, conducted a series of exercises to determine a POD by extrapolating from a figure in Baccarelli et al. (2008). Specifically, the USEPA determined the maternal serum concentration associated with neonatal TSH levels >5 μU/mL from a regression plot in Baccarelli et al. (2008) (reproduced in Figure 2). This plot presents regression findings for the crude analyses; results of the multivariate analyses in which confounding was considered result in a different regression slope (β = 0.75) (i.e., a different POD would be derived using the relationship that adjusted for confounding). The study authors did not provide sufficient data to determine a POD using the adjusted data. Because the USEPA has recognized the importance of confounding as part of the inclusion criteria, not using adjusted data lends uncertainty to the RfD.

### 4.4 Independent analysis demonstrating broad confidence interval around points of departure

No estimates of variability around the POD were considered by USEPA (2012). Therefore, an independent analysis was conducted to characterize the 95% confidence bounds around the serum concentration used for the POD. Data points were reconstructed from Figure 2A in Baccarelli et al. (2008) using WebPlotDigitizer version 4.1 (49 of the reported 51 data points were identified). A linear regression model was fit to the reconstructed data (log‐transformed neonatal TSH vs. log‐transformed maternal lipid‐adjusted serum TCDD), using R (R Core Team, 2018). This model was then used to make an inverse prediction of log maternal TCDD corresponding to a specified log neonatal TSH, along with the corresponding 95% confidence interval. The inverse prediction and confidence interval were calculated using the “chemCal” R package (Ranke, 2015), which adapts the calculation method of Massart et al. (1997). The prediction and 95% confidence interval bounds were then exponentiated to back‐transform them to the natural scale. It is recognized that this exercise was limited to the log‐transformed data as this was the form presented by the original authors; however, uncertainty could be even further characterized using non‐log transformed data (recognizing that this approach would have additional complexity associated with non‐linearity when assessing a confidence interval on an inverse relationship).

The resulting confidence interval was very broad (~40 ppt, >100 000 ppt) and reflects the high variability in the relationship, small sample size (*n* = 51), relatively small proportion of measurements in the higher TCDD ranges (>50 ppt) and non‐linear bivariate relationship (note the log‐transformed scales in the graphic provided by the original authors). The large confidence boundaries demonstrate a low level of precision in the estimate (and greater uncertainty).

### 4.5 Uncertainty in the points of departure based on lack of clinical significance (i.e., lack of adversity) of thyroid‐stimulating hormone measurements in Baccarelli et al. (2008)

The increased TSH was identified as an indicator of reduced circulating thyroxine (T_4_) levels, which could eventually lead to neurological deficiencies. More specifically, increased TSH was characterized as adverse based on its use as a screening indicator for follow‐up examination to rule in or rule out the presence of permanent congenital hypothyroidism (CH) (USEPA, 2012; WHO, 1994). However, the diagnosis of CH is significantly more complex than the assessment of a single screening level TSH sample (American Association of Pediatrics, 2006). Furthermore, screening for CH is complex. In a clinical setting, infants with TSH above a designated screening level (typically >10 μU/mL, discussed below) undergo confirmatory testing involving serum TSH and free T_4_. Those with both *persistent* serum TSH >10 μU/mL *and* normal or low free T_4_ are considered hypothyroid. Such cases are typically also accompanied by clinical evaluation, biochemical determinations, thyroid scintiscan and/or neck ultrasound (Corbetta et al., 2009). Similarly, Baccarelli et al. (2008) described the need for further testing in participants in the cohort; additional laboratory and clinical investigations were conducted on participants with β‐TSH >10 μU/mL. Two of eight (25%) individuals who were screened received a final diagnosis of permanent CH (discussed further below).

Thus, interpreting neonatal TSH results establishing adverse, e.g., the presence of CH, is complicated by the need to establish multiple lines of evidence, including repeat TSH measurements, additional thyroid hormone assessments (e.g., T_4_ and tri‐iodothyronine) and longer‐term medical monitoring (Saleh et al., 2016). Further, characterization of a cutoff of 5 μU/mL TSH as a LOAEL is not supported by the clinical literature. Rather, 5 μU/mL, and even concentrations up to 10 μU/mL, constitutes POD that represents a NOAEL, thus eliminating the need for the 10× UF.

### 4.6 Uncertainty in the points of departure due to lack of consideration of factors impacting variability in thyroid‐stimulating hormone levels (bias in outcome assessment)

One of the reasons it is difficult to rely on single measurements of TSH alone as a marker of adversity is the natural variability of TSH in infants. β‐TSH levels in infants have been reported to range from 1.7 to 9.1 μU/mL in children 2‐20 weeks of age (American Academy of Pediatrics [AAP], 2006). TSH levels are both transient and dependent on a number of factors (e.g., mode of birth, iodine deficiency) (Parks et al., 2010; Chanoine et al., 1988; LaFranchi, 2011). In the majority of cases, transient elevations of TSH can be attributed to many contributing factors other than permanent CH (Chanoine et al., 1988; Colon & Alonso‐Fernandez, 2011; LaFranchi, 2011). It is well known that iodine deficiency can induce transient CH (Parks et al., 2010), a condition that is neither related to TCDD exposure nor necessarily associated with adverse health effects, because it is easily corrected with iodine replacement therapy until normal iodine balance is achieved.

In addition, variability in neonatal TSH levels can be related directly to the postnatal timing of sample collection. TSH levels dramatically increase at delivery, possibly in response to neonatal cooling. These values typically reach 70 μU/mL within 30 minutes of birth, followed by a decline to about 20 and 10 μU/mL, at 24 and 48 hours postpartum, respectively. TSH values (both the mean and 97.5th percentile) measured between 1 and 7 days following birth decrease by at least threefold during this period (Lott, Sardovia‐Iyer, Speakman, & Lee, 2004). This variability related to the postnatal timing of sample collection was demonstrated in a study of 161 244 births based on the AutoDelfia method (the same TSH method reported by Baccarelli et al., 2008). All of the TSH samples (*n* = 51) from Baccarelli et al. (2008) are within the range of values reported by Lott et al. (2004) for the 72‐hour postpartum time interval. Thus, variability in the TSH values reported in Baccarelli et al. (2008) may largely reflect the expected variation due to sampling time. Importantly, Baccarelli et al. (2008) do not report the specific time of sample collection—a factor that precluded use of the study by EFSA (2018b) in their recent review. Specifically, EFSA reported that missing data on the timing of blood TSH made the quantitative association between TCDD levels and TSH uncertain.

The type of delivery also impacts postnatal TSH levels. For example, a study of 1859 neonates tested on day 3, also using the AutoDelfia TSH method, observed that 4.3% of neonates delivered vaginally were found to exceed the 5 μU/mL cutoff, compared with an exceedance rate of 7.1% for neonates born via Caesarean section (McElduff, McElduff, Wiley, & Wilcken, 2005). Although Baccarelli et al. (2008) accounted for the type of delivery in the multivariate analysis and tabular summaries, the POD was based on the graphical summary using raw data for *n* = 51 mother‐infant pairs, not adjusted for covariates. Thus, the POD also does not account for the potential impact of the type of delivery.

### 4.7 Uncertainty in the points of departure associated with using a screening level of 5 μU/mL as a threshold for adverse effects (bias in outcome assessment)

The >5 μU TSH/mL value used by the USEPA as a threshold for adverse effects is not a level associated with adverse effects in individuals. The USEPA acknowledged this uncertainty, stating that “the exact relationship between TSH increases and adverse neurodevelopmental outcomes is not well defined” (USEPA, 2012, pp. 4‐57). The threshold of 5 μU/mL is a screening value proposed by WHO (1994), but only as an indicator of iodine deficiency. Because iodine is essential for the synthesis of thyroid hormones, TSH levels can directly reflect the availability and adequacy of thyroid hormone. In 1994, WHO described TSH levels as the best diagnostic test for determining hypothyroidism, although also clarifying that TSH levels are a *screening* measure. A screening level serves as a recall threshold for the initial neonatal β‐TSH measurement that triggers further diagnostic measures. When describing how to interpret levels, WHO identifies a recall threshold of 10‐15 μU/mL to screen for CH, whereas 5 μU/mL is described as a screening level that may be appropriate for identifying iodine‐replete populations in epidemiologic studies.

In the Lombardy region (where Seveso is located), 10 μU/mL served as the recall threshold at the time of the Baccarelli et al. (2008) study (Corbetta et al., 2009). In addition, in Baccarelli et al. (2008), the study authors themselves used 10 μU/mL as their measure of adversity. Other entities have also established similar comparison values. For example, the diagnostic guidance from the AAP provides a helpful perspective, noting that “most physicians would consider a persistent basal TSH concentration higher than 10 μU/mL (after the first 2 weeks of age) to be abnormal. … A TSH range of 1.7 to 9.1 μU/mL has been reported for children 2 to 20 weeks of age” (AAP, 2006, p. 2295). The Laboratory Support for the Diagnosis of Monitoring of Thyroid Disease of the National Academy of Clinical Biochemistry considers <10 μU/mL a no‐further‐action level.

The majority of the 1014 neonates evaluated by Baccarelli et al. (2008) had β‐TSH levels <10 μU/mL. Only eight of the study participants (across the entire study, including comparisons) had β‐TSH >10 μU/mL (the recall threshold in Lombardy), and following assessment in recall tests, five were found to have β‐TSH <5 μU/mL (did not undergo further testing), and only two were eventually confirmed to have CH (status of eighth participant following recall tests unknown). None of the other neonates were followed further, which is the usual practice; therefore, the CH rate for children in both the exposed and comparison populations is unknown. This includes uncertainty regarding children who did not screen in (i.e., false negatives). Additionally, the authors reported that the proportion of newborns with β‐TSH >5 μU/mL was 2.8% in the reference area, a finding similar to that of WHO, which anticipated ~3% of infants in iodine‐replete populations to exceed this level, based simply on natural variability (Baccarelli et al., 2008, p. 1135).

If the serum concentration had been based on the TCDD level at which the dose‐response function in the regression analysis presented by Baccarelli et al. (2008) reaches 10 μU/mL, the resulting serum concentration would be 1513.56 ppt, with a lower 95% confidence boundary of 151.36 ppt and an upper 95% confidence boundary >10 000 000 ppt, i.e., use of 10 μU/mL—the standard “threshold” implemented clinically—results in serum concentrations that are orders of magnitude higher than that estimated using the 5 μU/mL threshold.

## 5 UNCERTAINTY IN THE PHARMACOKINETIC MODELING USED TO DEVELOP THE POINTS OF DEPARTURE

In selecting a model, the USEPA considered several published models for TCDD (Aylward, Brunet, Carrier, et al., 2005; Emond, Birnbaum, & DeVito, 2004; Emond, Birnbaum, & DeVito, 2006; Emond, Michalek, Birnbaum, & DeVito, 2005), although, ultimately, the agency used the model developed by Emond et al. (2006), with modifications. It should be noted that the USEPA rigorously evaluated potential models and, in doing so, reported that the simulation results of serum lipid or liver concentrations varied up to a factor of 7 (USEPA, 2012), thus recognizing the potential uncertainties inherent to the use of such models. The uncertainty in the model has been demonstrated in comments provided to the USEPA (Science Advisory Council [SAC], 2010), as well as more broadly in the literature (e.g., Aylward, Collins, Bodner, Wilken, & Bodnar, 2013).

The consequence of the half‐life overprediction (i.e., too long) by physiologically based pharmacokinetic models, relative to empirically measured elimination behavior in humans, is that chronic daily doses associated with serum concentrations <100 ppt will be underestimated by the models; hence, the dose predicted to produce a 100‐ppt serum concentration will be too low, i.e., the intake dose to achieve the lipid‐adjusted serum concentration (LASC) would be higher. This issue would also result in an underestimate of intakes required to achieve somewhat higher body burdens due to the underestimation of elimination rates in the lower concentration range of the accumulation process.

Additionally, children, the sensitive population represented by the Seveso data from both Mocarelli et al. (2008) and Baccarelli et al. (2008), eliminate TCDD faster than adults do (Milbrath et al., 2009). The intestinal lipid clearance and concomitant elimination of TCDD is much faster in infants and children than in adults, but the Emond model does not accurately reflect this accelerated clearance. Therefore, the model underpredicts the intake rate associated with the target serum lipid concentrations by a factor of ≥2 (SAC, 2010). Taken together, the physiologically based pharmacokinetic model employed to develop estimates of intake dose is associated with uncertainty; uncertainty suggesting that the intake doses estimated by the model are low.

## 6 UNCERTAINTIES RELATED TO LACK OF CONSIDERATION OF TOXIC EQUIVALENCY AND IMPACTS ON DOSE ESTIMATES

Another major source of uncertainty associated with the USEPA RfD is lack of consideration of the total TEQ serum concentration. In identifying the serum concentration to be used in the development of the POD, the USEPA focused solely on TCDD. This is problematic, given the practical application of the RfD in assessing health risks, i.e., when evaluating risk from DLCs, it is commonly accepted, as well as recommended by the USEPA, that the WHO TEQ method be used (USEPA, 2010a, 2010b). The TEQ method accounts for additive effects of all DLCs by assigning toxic equivalency factors to each of the DLCs and then summing them to obtain a total TEQ concentration (Van den Berg et al., 2006). This method is intended to address the fact that the DLCs act through a common mechanism of action that involves binding the aryl hydrocarbon receptor to induce a similar spectrum of adverse effects. Thus, any potential adversities observed would be a result of collective DLC activity. When considering such effects, a serum concentration based on TEQ would form the basis of the POD (rather than a serum concentration based on TCDD alone). Failure to account for the total TEQ concentration results in an overestimation of the potency of TCDD.

**Figure 3:**
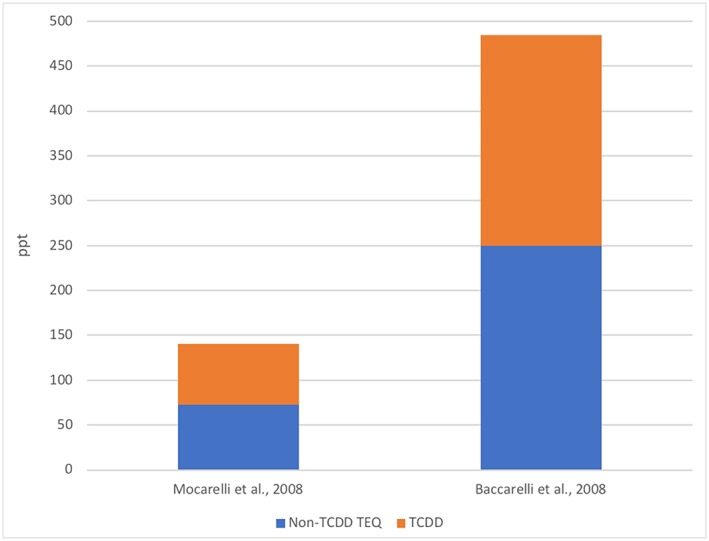
Contribution of TCDD to overall TEQ, as determined by USEPA (2012); demonstrates that TCDD potency is overestimated by not considering TEQ. TCDD, 2,3,7,8‐tetrachlorodibenzo‐p‐dioxin; TEQ, toxic equivalency [Colour figure can be viewed at wileyonlinelibrary.com]

The USEPA cited a lack of relevant background TEQs in the Seveso populations as part of the rationale for not incorporating background exposures to dioxins into the POD. However, the contribution of other DLCs was significant to the overall TEQs in both of the co‐critical studies (Baccarelli et al., 2008; Mocarelli et al., 2008) (Figure 3). Baccarelli et al. (2008) report that the mean maternal TCDD level was 18.9 ppt, whereas the mean concentration based on TEQ (including polychlorinated dibenzodioxins and furans [PCDD/Fs] and dioxin‐like polychlorinated biphenyls) was 41.8 ppt, i.e., TCDD accounted for less than half of the total TEQ (Figure 3). Mocarelli et al. (2008, p. 73) did not report serum TEQ, although they stated, “If TCDD acts in concert with other dioxin‐like chemicals in affecting sperm quality, the total dioxin toxic equivalency (TEQ) should be considered.” This text is followed by the citation of a publication reporting on serum concentrations in women from Seveso, which reported serum concentrations of ~100 ppt, of which ~20 ppt is from TCDD (Eskenazi et al., 2004). These data discussed by Mocarelli et al. (2008) would suggest that TCDD potentially accounts for only 20% of the TEQ. Thus, there is direct evidence that, for both co‐critical studies, TCDD represents only a portion of the overall TEQ. The impact is that using TCDD concentrations alone ignores the contribution of other DLCs and results in a significant overestimation of TCDD potency in eliciting any purported adverse effects.

The TEQ concept was acknowledged by the USEPA as part of the agency's sensitivity analysis of the RfD (discussed below): “Because DLCs are presumed to act in the same manner as TCDD (for AhR induction and subsequent effects), the magnitude of the background DLC exposure is an important concern in establishing the POD” (USEPA, 2012, pp. 4‐77). For this reason, the USEPA considered the contribution of other PCDD/Fs or their potential contribution to the overall dioxin TEQ, but the other PCDD/Fs were not included in the final derivation of the RfD. A series of calculations and modeling exercises were used to evaluate the influence of TEQ (as well as selected other parameters). For this evaluation, it was assumed that TCDD was 10% of the TEQ. For Mocarelli et al. (2008), the total TEQ associated with the median TCDD of 68 ppt was estimated to be 140.1 ppt TEQ (72.5 non‐TCDD TEQ); for Baccarelli et al. (2008), the total TEQ associated with the maternal serum concentration related to infant TSH >5 μU/mL was 485 ppt. The USEPA concluded that the consideration of background exposure (i.e., TEQ vs. TCDD alone) was the most influential variable included in the sensitivity analysis, thus demonstrating the importance of considering total TEQ vs. TCDD only.

## 7 QUALITATIVE CHARACTERIZATION OF OVERALL UNCERTAINTY

**Figure 4:**
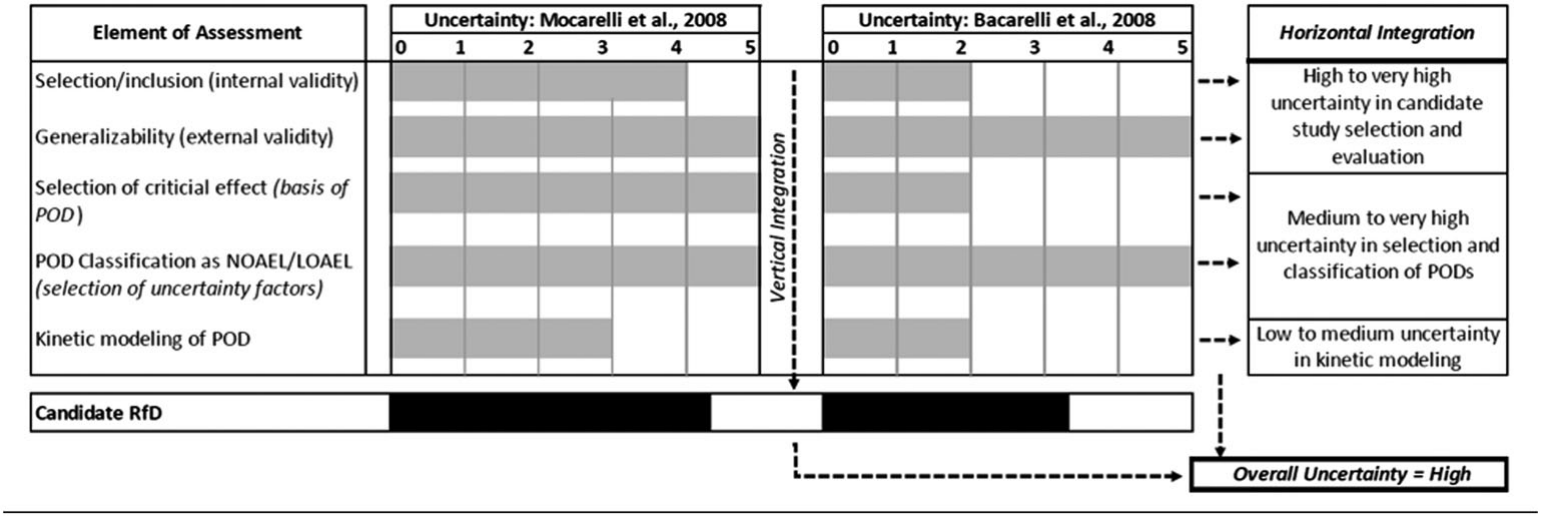
Horizontal and vertical integration of uncertainty per National Academies of Science, Engineering, and Medicine (2014) recommendations. LOAEL, lowest‐observed‐adverse‐effect level; NOAEL, no‐observed‐adverse‐effect level; POD, points of departure; RfD, reference dose

As shown in Figure 4, when the co‐critical studies were deconstructed as described above, the integration of each stage resulted in a “high to very high” level of uncertainty in candidate study selection based on internal validity as it relates to inclusion based on study quality and reliability (e.g., lack of control for confounding, uncertainties in measures of the outcome, etc.). Both studies had a high level of uncertainty related to generalizability of exposure‐response relationships observed from the Seveso incident to general‐population exposure‐response relationships (noting that studies in experimental animals support the observation of differential kinetics and outcomes following high‐dose exposures). There was a “medium to very high” level of uncertainty in selection, derivation and classification of PODs; this categorization was driven by lack of statistical or clinical significance, subsequent classification of the PODs as LOAELs (vs. NOAELs) and a lack of consideration of TEQ.

When integrated vertically (i.e., when considering all the elements evaluated collectively for each study), the resulting overall level of uncertainty was “high.” This designation was determined simply because of the high level of uncertainty in each of the parameters considered resulting in a collective designation of high uncertainty. When the co‐critical studies were compared, the uncertainty associated with Mocarelli et al. (2008) was determined substantially higher than that of Baccarelli et al. (2008), owing primarily to limitations in the validity of the evaluation of single, altered sperm concentrations in men from the Seveso cohort, combined with the lack of statistical and clinical significance of the data selected to develop the POD. When uncertainty in candidate values from both studies was combined, the overall uncertainty was considered “high.”

## 8 QUANTITATIVE CHARACTERIZATION OF OVERALL UNCERTAINTY

The use of alternative assumptions identified during the deconstruction of the co‐critical studies allows for the determination of alternative RfD values that could reasonably be considered. Quantitative characterization of alternatives focused on aspects such as statistical significance, clinical significance, TEQ (vs. TCDD only) and uncertainties in the kinetic modeling. However, owing to the very high level of uncertainty (and thus lack of reliability for the purposes of risk assessment) in the Mocarelli et al. (2008) study, quantitative uncertainty analyses were conducted only for Baccarelli et al. (2008).

**Figure 5:**
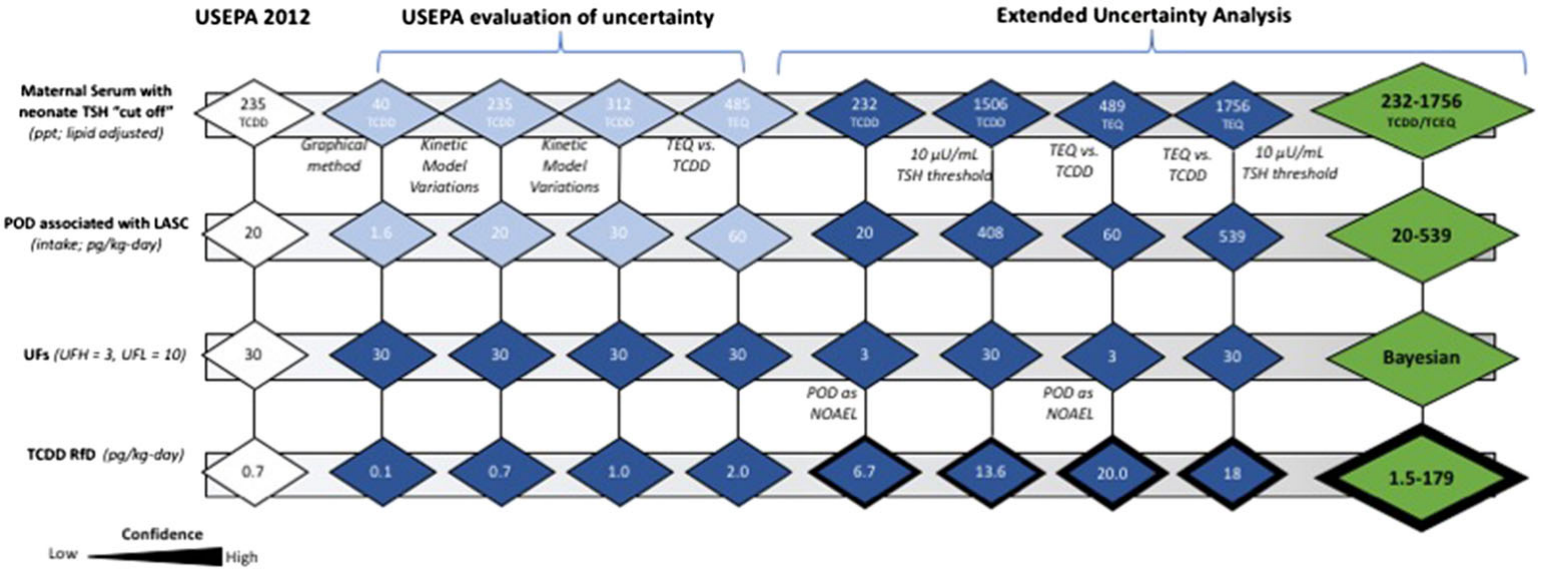
Sensitivity tree demonstrating the range of plausible RfD values from Baccarelli et al. (2008). Light blue shade indicates parameters evaluated by USEPA, dark blue indicates parameters identified via deconstruction of the RfD, and green symbols indicate combined uncertainty evaluation integrating Bayesian assessment of UFs. Symbol outline indicates level of confidence. LASC, lipid‐adjusted serum concentration; NOAEL, no‐observed‐adverse‐effect level; POD, points of departure; RfD, reference dose; TCDD, 2,3,7,8‐tetrachlorodibenzo‐p‐dioxin; TCEQ, Texas Commission on Environmental Quality; TEQ, toxic equivalency; TSH, thyroid‐stimulating hormone; UFs, uncertainty factors; USEPA, US Environmental Protection Agency

The quantitative uncertainty analysis conducted for Baccarelli et al. (2008) involved combining parameters used by USEPA in their sensitivity analysis of key interpretive decisions associated with exposure and kinetic modeling (USEPA, 2012), along with alternative POD values and LOAEL/NOAEL characterizations. With respect to uncertainty parameters from USEPA (2012), selected scenarios from the sensitivity trees were used for demonstrative purposes. Because the USEPA sensitivity analysis involved only derivation of alternative PODs (Figure 5; light blue shading), we applied the UFs to derive the alternative RfD values (Figure 5; dark blue shading). The USEPA characterizations integrated TEQ for some scenarios. For example, TCDD‐only estimates ranged from 0.00161 to 0.0303 ng/kg/day for Baccarelli et al. (2008), whereas PODs based on TEQ were 0.0180 and 0.0593 ng/kg/day. Other scenarios represent variations in the maternal serum (and the POD) using alternative kinetic modeling assumptions.

Additional scenarios presented in Figure 5 involve alternative combinations that incorporated: (1) characterization of the POD as a NOAEL vs. LOAEL (results in composite UF of 3 instead of 30); (2) addition of TEQ; and (3) maternal serum concentrations using a TSH cutoff of 10 μU/mL (vs. 5 μU/mL). Thus, when viewed from left to right, the sensitivity tree (Figure 5) displays alternative candidate RfDs using the Baccarelli et al. (2008) dataset by increasing confidence.

As per the recommendations from National Academies of Science, Engineering, and Medicine (2014), uncertainty was also evaluated using a Bayesian approach that combined distribution of POD values, as well as UFs (Figure 5; green shaded symbols). POD values were based on the regression from Baccarelli et al. (2008) for neonatal TSH vs. maternal TCDD and maternal TEQ (Data S2; see Supporting Information); LASC values corresponding to two threshold values of neonatal TSH (5 and 10 μU/mL) were determined for TCDD and TEQ. The POD values (serum concentrations) were converted into equivalent intake dose PODs and then to RfDs using a Bayesian approach to applying UFs. This approach, endorsed by National Academies of Science, Engineering, and Medicine (2014) and discussed by Simon et al. (2016), involves treating the UF as a random variable (vs. a fixed variable) obeying a log‐normal distribution, thus resulting in a distribution of relative potency values. The assumption of a log‐normal distribution for the UF is generic, taken in the absence of detailed data that could define an empirical distribution for the UF (Simon et al., 2016). In the language of Bayesian statistics, the log‐normal distribution for the UF is a previous distribution, as is any distribution for the POD: the resulting RfD distribution thus represents an “induced prior.”

**Figure 6:**
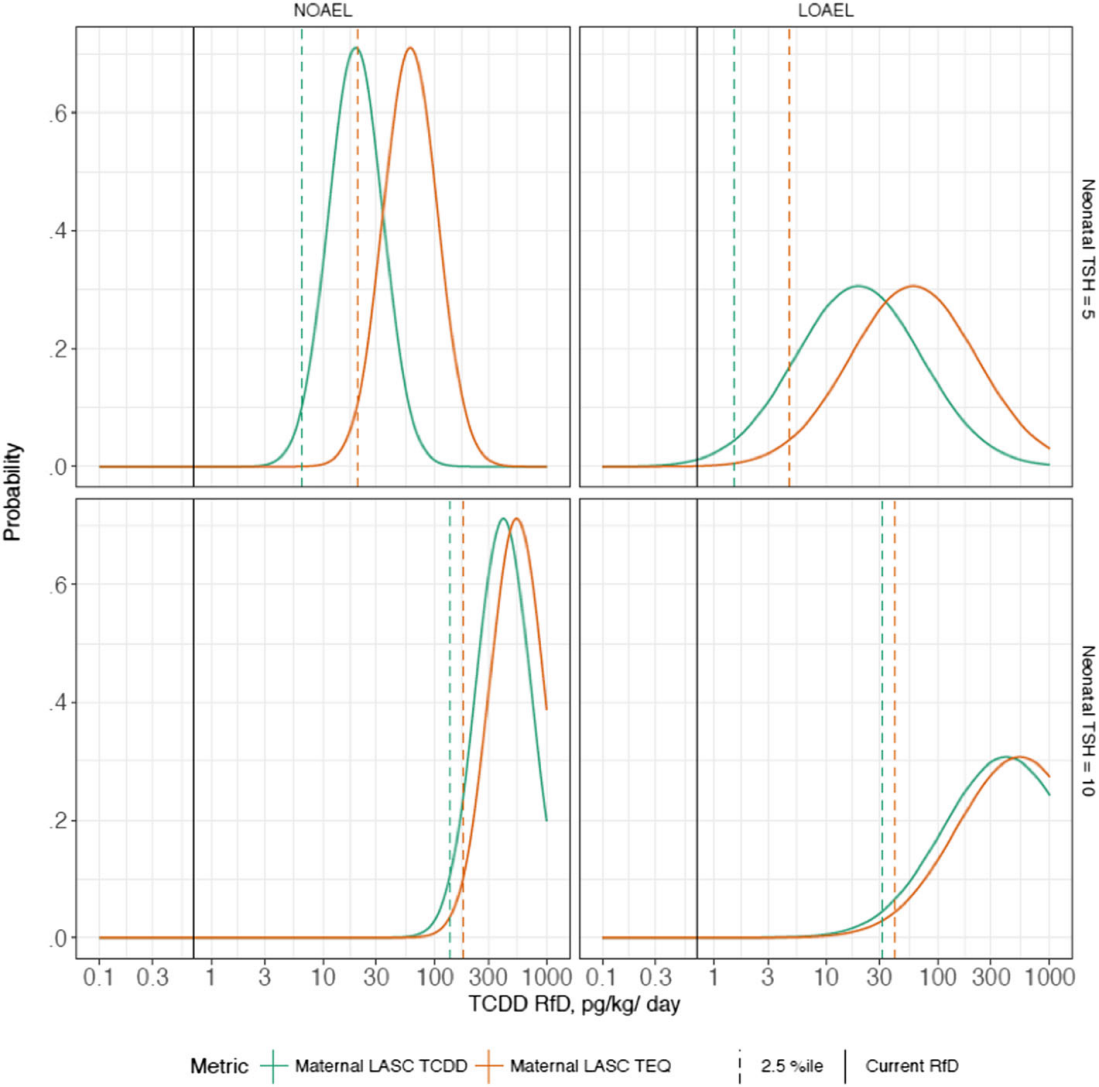
RfDs derived using Bayesian approach to incorporating uncertainty factors. Right column: RfD derived treating POD as LOAEL; left column: RfD derived treating POD as NOAEL. Top row: RfD derived for a neonatal TSH threshold of 5 μU/mL; bottom row: RfD derived for a neonatal TSH threshold of 10 μU/mL. Blue solid lines: probability distributions for RfD derived using POD expressed as maternal LASC TCDD. Red solid lines: probability distributions for RfD derived using POD expressed as maternal LASC TEQ. Blue dashed vertical lines: 2.5th percentile of RfD derived from POD as maternal LASC TCDD (lower bound of two‐tailed 95% confidence interval on RfD). Red dashed vertical lines: 2.5th percentile of RfD derived from POD as maternal LASC TEQ (lower bound of two‐tailed 95% confidence interval on RfD). Black solid vertical lines: Current RfD (0.7 pg/kg/day). LOAEL, lowest‐observed‐adverse‐effect level; LASC, lipid‐adjusted serum concentration; NOAEL, no‐observed‐adverse‐effect level; POD, points of departure; RfD, reference dose; TCDD, 2,3,7,8‐tetrachlorodibenzo‐p‐dioxin; TEQ, toxic equivalency; TSH, thyroid‐stimulating hormone

In this evaluation, each POD could be treated either as an NOAEL (combined with an UF of 3) or as an LOAEL (combined first with an UF of 10, then with an UF of 3). As a result, eight different distributions for RfD were derived, for the different combinations of POD exposure metric (TCDD or TEQ), POD neonatal TSH threshold (5 or 10), and POD type (NOAEL or LOAEL). These eight distributions are shown in Figure 6 (colored curves). For each distribution, the 2.5th percentile was taken as a conservative, lower‐bound estimate for an RfD (marked with vertical colored dashed lines in Figure 6). The 2.5th percentile RfD estimates from the eight distributions range from ~1.5 to 179 pg/kg/day (Figures 5 and 6).

The position of the current RfD on each of the eight distributions was computed (marked with vertical black solid lines in Figure 6). The current RfD is at the extreme lower tail of all eight distributions, occurring at percentiles ranging from 0.5 (i.e., 99.5% of values are higher than the current RfD) down to 1e‐30 (i.e., 99.999 … % of values are higher than the current RfD). This demonstrates the highly conservative nature of the current RfD.

## 9 DISCUSSION AND CONCLUSIONS

Using approaches for characterizing uncertainty, as recommended by National Academies of Science, Engineering, and Medicine (2014), Beck et al. (2016) and WHO (2017), and building on the initial efforts of USEPA (2012), we have assessed uncertainties in the development of the RfD for the TCDD. We have demonstrated that of the two co‐critical studies, Baccarelli et al. (2008) is more reliable. Key elements forming the basis of this conclusion included limitations in the process for selection, external and internal validity of the individual studies, and selection and derivation of the POD using data from the individual studies. Quantitative integration of the uncertainties resulted in plausible RfDs ranging from ~1.5 to 179 pg/kg/day. Furthermore, application of Bayesian techniques for the assessment of UFs demonstrated that the current RfD is at the lower end of all possible distributions described herein.

Several qualitative observations were noted regarding elements of the Seveso studies that contribute significantly to the overall uncertainty. This included a high level of uncertainty related to external validity (i.e., lack of generalizability of acute, high‐dose exposures to typical population exposures), and a high level of uncertainty related to the selection of and confidence in the POD, given that high‐dose mechanisms have been documented for both outcomes (increased TSH and decreased sperm) (Bell et al., 2007; Black et al., 2012; Budinsky, LeCluyse, Ferguson, Rowlands, & Simon, 2010; Connor & Aylward, 2006; Foster et al., 2010; Goodman et al., 2010). Extrapolation of daily intakes associated with serum concentrations resulting from an acute, high‐dose exposure were also associated with uncertainty due to the concentration‐ and age‐dependent kinetics of TCDD (Aylward, Brunet, Starr, et al., 2005). Further complicating this extrapolation is the role of other DLCs (i.e., TEQ). Notably, even when using experimental studies of animals exposed to TCDD as the basis of the benchmark, JECFA (2002) (joint FAO/WHO expert committee on food additives) accounted for the differences in daily exposure associated with long‐term vs. acute exposure, as well as for background TEQ body burdens.

Another common theme was related to the selection of each study as being representative of the respective outcome, i.e., Was each study reasonably representative of the body of evidence? To address this issue, a systematic review of each outcome would need to be conducted, and the consistency in the direction of the findings, dose‐response relationships, magnitude, etc., of the body of evidence would be determined. Such an approach would include evaluating and ranking individual studies according to methodological rigor—an aspect that is well recognized, e.g., in assessing the potential for an agent to affect male fertility based on semen parameters, or the potential for a single measurement of increased TSH linked to adverse outcomes. It is thus notable that between submission of this current manuscript and final acceptance, EFSA released a risk assessment that incorporated a systematic review in developing toxicity values for TCDD (EFSA, 2018b). Many of the methodological validity questions relating to Mocarelli et al. (2008) and Baccarelli et al. (2008) discussed herein were also noted by EFSA. Also notable, these studies were not selected by EFSA as candidate studies to derive a toxicity value. Further supporting the importance of characterizing uncertainty, EFSA (2018b) also included a formal uncertainty assessment that included consideration of uncertainty in hazard identification and characterization (including both human and animal studies), dose‐response assessment, benchmark dose modeling, toxicokinetic modeling and risk characterization. Based on such, and the for the larger body of evidenced considered by EFSA (2018b), they also concluded that the impact of the uncertainties on the risk assessment of the dioxins was “high.”

On a broader scale, it is apparent that the increased use of epidemiological data in chemical risk assessment—while preferred over animal data—requires additional guidance and potential refinements to methods traditionally used for animal data. This scenario can be equated to evaluation of an animal study based on Good Laboratory Practice or guideline methods, or other measures of study quality (i.e., use of established standards to determine the confidence and sensitivity of an epidemiological study in characterizing the exposure‐response). Such efforts range from how data are selected and evaluated for quality (e.g., risk of bias) to how data from studies are extracted and used in quantitative assessments (National Academies of Science, Engineering, and Medicine, 2006; Van Landingham et al., 2016). The NAS specifically noted that, in cases where the critical studies used for the development of reference values were the only human studies with sufficient exposure and dose‐response data to support risk estimation, study weaknesses still must be considered, and that such studies undergo a risk of bias assessment (National Academies of Science, Engineering, and Medicine, 2011). Both Mocarelli et al. (2008) and Baccarelli et al. (2008) had methodological limitations that were objectively characterized using an informal assessment of internal and external validity. This, again, reinforces the positive direction of integrating a systematic review (Bahadori & Thayer, 2018), including evaluation of the study validity (and risk of bias), into the IRIS process, although it also highlights the need for continued refinement of existing tools (e.g., “APROBA,” a tool being considered by IRIS to aid in characterization of uncertainty does not accommodate human data) (Blessinger & Bussard, 2018). Also of note, a systematic review of sperm endpoints is currently under way (Urban et al., 2018).

The range of plausible RfDs derived in this assessment highlights the importance of characterizing the uncertainties more fully, both in the underlying database and in the approaches used to establish the RfD; toxicity values cannot be viewed as “bright lines.” As described by the WHO, uncertainty analyses provide an opportunity to inform more transparently the confidence we can place in toxicological risk projections and estimates of the relationship between dose and health effect, thereby facilitating choices of preventative measures taken by risk managers (WHO, 2017), i.e., characterizing and communicating uncertainty allows risk assessors to communicate better the confidence and degree of health protection inherent in assessments that include specific toxicity values. It is anticipated that the range of RfDs (~1.5 to 179 pg/kg/day), along with characterization of confidence in those values, will improve risk assessments of DLCs and provide important information to risk managers.

### CONFLICT OF INTERESTS

This work was funded by Dow Chemical Company.

## Supporting information

Data S1
Supporting information

Data S2
Supporting information

## References

[jat3814-bib-0001] AAP (2006). Update of newborn screening and therapy for congenital hypothyroidism. Pediatrics, 117(6), 2290–2303. 10.1542/peds.2006-0915 16740880

[jat3814-bib-0002] Aylward, L. L. , Brunet, R. C. , Carrier, G. , Hays, S. M. , Cushing, C. A. , Needham, L. L. , … Mocarelli, P. (2005). Concentration‐dependent TCDD elimination kinetics in humans: toxicokinetic modeling for moderately to highly exposed adults from Seveso, Italy, and Vienna, Austria, and impact on dose estimates for the NIOSH cohort. Journal of Exposure Analysis and Environmental Epidemiology, 15(1), 51–65. 10.1038/sj.jea.7500370 15083163

[jat3814-bib-0003] Aylward, L. L. , Brunet, R. C. , Starr, T. B. , Carrier, G. , Delzell, E. , Cheng, H. , & Beall, C. (2005). Exposure reconstruction for the TCDD‐exposed NIOSH cohort using a concentration‐ and age‐dependent model of elimination. Risk Analysis, 25(4), 945–956. 10.1111/j.1539-6924.2005.00645.x 16268942

[jat3814-bib-0004] Aylward, L. L. , Collins, J. J. , Bodner, K. M. , Wilken, M. , & Bodnar, C. M. (2013). Elimination rates of dioxin congeners in former chlorophenol workers from Midland, Michigan. Environmental Health Perspectives, 121(1), 39–45. 10.1289/ehp.1205544 23063871 PMC3552814

[jat3814-bib-0005] Baccarelli, A. , Giacomini, S. M. , Corbetta, C. , Landi, M. T. , Bonzini, M. , Consonni, D. , … Bertazzi, P. A. (2008). Neonatal thyroid function in Seveso 25 years after maternal exposure to dioxin. PLoS Medicine, 5, e161 10.1371/journal.pmed.0050161 18666825 PMC2488197

[jat3814-bib-0006] Bahadori, T. , & Thayer, K. A. (2018). National Academy of Sciences Committee to review advances made to the IRIS process. Paper presented at the Review of the Advances Made to the IRIS Process: A Workshop, Washington, DC. http://nas‐sites.org/dels/files/2018/01/AdIRIS‐15.pdf

[jat3814-bib-0007] Beck, N. B. , Becker, R. A. , Erraguntla, N. , Farland, W. H. , Grant, R. L. , Gray, G. , … Dourson, M. L. (2016). Approaches for describing and communicating overall uncertainty in toxicity characterizations: US Environmental Protection Agency's Integrated Risk Information System (IRIS) as a case study. Environment International, 89–90, 110–128. 10.1016/j.envint.2015.12.031 26827183

[jat3814-bib-0008] Bell, D. R. , Clode, S. , Fan, M. Q. , Fernandes, A. , Foster, P. M. , Jiang, T. , … White, S. (2007). Toxicity of 2,3,7,8‐tetrachlorodibenzo‐p‐dioxin in the developing male Wistar (Han) rat. I: No decrease in epididymal sperm count after a single acute dose. Toxicological Sciences, 99(1), 214–223. 10.1093/toxsci/kfm140 17545212

[jat3814-bib-0009] Bell, D. R. , Clode, S. , Fan, M. Q. , Fernandes, A. , Foster, P. M. , Jiang, T. , … White, S. (2010). Interpretation of studies on the developmental reproductive toxicology of 2,3,7,8‐tetrachlorodibenzo‐p‐dioxin in male offspring. Food & Chemical Toxicology, 48(6), 1439–1447. 10.1016/j.fct.2010.04.005 20388530 PMC2923583

[jat3814-bib-0010] Bichteler, A. , Wikoff, D. S. , Loko, F. , & Harris, M. A. (2017). Estimating serum concentrations of dioxin‐like compounds in the US population effective 2005‐2006 and 2007‐2008: A multiple imputation and trending approach incorporating NHANES pooled sample data. Environment International, 105, 112–125. 10.1016/j.envint.2017.05.003 28527750

[jat3814-bib-0011] Black, M. B. , Budinsky, R. A. , Dombkowski, A. , Cukovic, D. , LeCluyse, E. L. , Ferguson, S. S. , … Rowlands, J. C. (2012). Cross‐species comparisons of transcriptomic alterations in human and rat primary hepatocytes exposed to 2,3,7,8‐tetrachlorodibenzo‐p‐dioxin. Toxicological Sciences, 127(1), 199–215. 10.1093/toxsci/kfs069 22298810

[jat3814-bib-0012] Blessinger, T. , & Bussard, D. (2018). Quantitative evaluation of uncertainty: APROBA and beyond. Paper presented at the review of advances made to the IRIS process: A workshop, Washington, DC. http://nas‐sites.org/dels/events/review‐of‐advances‐made‐to‐the‐iris‐process‐a‐workshop/iris‐workshop‐presentationsmaterials/

[jat3814-bib-0013] Budinsky, R. A. , LeCluyse, E. L. , Ferguson, S. S. , Rowlands, J. C. , & Simon, T. (2010). Human and rat primary hepatocyte CYP1A1 and 1A2 induction with 2,3,7,8‐tetrachlorodibenzo‐p‐dioxin, 2,3,7,8‐tetrachlorodibenzofuran, and 2,3,4,7,8‐pentachlorodibenzofuran. Toxicological Sciences, 118(1), 224–235. 10.1093/toxsci/kfq238 20705892

[jat3814-bib-0014] Carlsen, E. , Petersen, J. H. , Andersson, A. M. , & Skakkebaek, N. E. (2004). Effects of ejaculatory frequency and season on variations in semen quality. Fertility & Sterility, 82, 358–366. 10.1016/j.fertnstert.2004.01.039 15302284

[jat3814-bib-0015] Chanoine, J. P. , Boulvain, M. , Bourdoux, P. , Pardou, A. , Van Thi, H. V. , Ermans, A. M. , & Delange, F. (1988). Increased recall rate at screening for congenital hypothyroidism in breast fed infants born to iodine overloaded mothers. Archives of Disease in Childhood, 63, 1207–1210. 10.1136/adc.63.10.1207 3196047 PMC1779012

[jat3814-bib-0016] Chiu, W. , Axelrad, D. , Dalaijamts, C. , Dockins, C. , Shao, K. , Shapiro, A. , & Paoli, G. (2018). Beyond the RfD: Broad application of a probabilistic approach to improve chemical dose‐response assessments for noncaner effects. Environmental Health Perspectives, 126(6), 067009 10.1289/EHP3368 29968566 PMC6084844

[jat3814-bib-0017] Connor, K. T. , & Aylward, L. L. (2006). Human response to dioxin: aryl hydrocarbon receptor (AhR) molecular structure, function, and dose‐response data for enzyme induction indicate an impaired human AhR. Journal of Toxicology and Environmental Health B Critical Reviews, 9(2), 147–171. 10.1080/15287390500196487 16613807

[jat3814-bib-0018] Corbetta, C. , Weber, G. , Cortinovis, F. , Calebiro, D. , Passoni, A. , Vigone, M. C. , … Persani, L. (2009). A 7‐year experience with low blood TSH cutoff levels for neonatal screening reveals an unsuspected frequency of congenital hypothyroidism (CH). Clinical Endocrinology (Oxford), 71(5), 739–745. 10.1111/j.1365-2265.2009.03568.x 19486019

[jat3814-bib-0019] EFSA (2018a). Guidance on uncertainty analysis in scientific assessments. EFSA Journal, 16(1), 39 10.2903/j.efsa.2018.5123 PMC7009727 32625671

[jat3814-bib-0020] EFSA (2018b). Risk for animal and human health related to the presence of dioxins and dioxin‐like PCBs in feed and food. EFSA Journal, 16(11), 5333 10.2903/j.efsa.2018.5333 PMC7009407 32625737

[jat3814-bib-0021] Emond, C. , Birnbaum, L. S. , & DeVito, M. J. (2004). Physiologically based pharmacokinetic model for developmental exposures to TCDD in the rat. Toxicological Sciences, 80(1), 115–133. 10.1093/toxsci/kfh117 15056810

[jat3814-bib-0022] Emond, C. , Birnbaum, L. S. , & DeVito, M. J. (2006). Use of a physiologically based pharmacokinetic model for rats to study the influence of body fat mass and induction of CYP1A2 on the pharmacokinetics of TCDD. Environmental Health Perspectives, 114(9), 1394–1400. 10.1289/ehp.8805 16966094 PMC1570044

[jat3814-bib-0023] Emond, C. , Michalek, J. E. , Birnbaum, L. S. , & DeVito, M. J. (2005). Comparison of the use of a physiologically based pharmacokinetic model and a classical pharmacokinetic model for dioxin exposure assessments. Environmental Health Perspectives, 113(12), 1666–1668. 10.1289/ehp.8016 16330344 PMC1314902

[jat3814-bib-0024] Eskenazi, B. , Mocarelli, P. , Warner, M. , Needham, L. , Patterson, D. G. Jr. , Samuels, S. , … Brambilla, P. (2004). Relationship of serum TCDD concentrations and age at exposure of female residents of Seveso, Italy. Environmental Health Perspectives, 112(1), 22–27. 10.1289/ehp.6573 14698926 PMC1241792

[jat3814-bib-0025] Fisch, H. , Ikeguchi, E. F. , & Goluboff, E. T. (1996). Worldwide variations in sperm counts. Urology, 48(6), 909–911. 10.1016/S0090-4295(96)00301-9 8973676

[jat3814-bib-0026] Foster, W. G. , Maharaj‐Briceno, S. , & Cyr, D. G. (2010). Dioxin‐induced changes in epididymal sperm count and spermatogenesis. Environmental Health Perspectives, 118(4), 458–464. 10.1289/ehp.0901084 20368131 PMC2854720

[jat3814-bib-0027] Goodman, J. E. , Kerper, L. E. , Boyce, C. P. , Prueitt, R. L. , & Rhomberg, L. R. (2010). Weight‐of‐evidence analysis of human exposures to dioxins and dioxin‐like compounds and associations with thyroid hormone levels during early development. Regulatory Toxicology & Pharmacology, 58(1), 79–99. 10.1016/j.yrtph.2010.04.008 20416351

[jat3814-bib-0028] Greene, J. F. , Hays, S. , & Paustenbach, D. (2003). Basis for a proposed reference dose (RfD) for dioxin of 1‐10 pg/kg/day: A weight of evidence evaluation of the human and animal studies. Journal of Toxicology & Environmental Health B Critical Reviews, 6(2), 115–159. 10.1080/10937400306470 12554432

[jat3814-bib-0030] Joint FAO/WHO Expert Committee on Food Additives (JECFA) (2002). Safety evaluation of certain food additives and contaminants. 57th Meeting of JECFA, 2001, Rome, Italy. World Health Organization https://apps.who.int/iris/handle/10665/42501

[jat3814-bib-0031] LaFranchi, S. H. (2011). Approach to the diagnosis and treatment of neonatal hypothyroidism. Journal of Clinical Endocrinology and Metabolism, 96, 2959–2967. 10.1210/jc.2011-1175 21976744

[jat3814-bib-0033] Lott, J. A. , Sardovia‐Iyer, M. , Speakman, K. S. , & Lee, K. K. (2004). Age‐dependent cutoff values in screening newborns for hypothyroidism. Clinical Biochemistry, 37(9), 791–797. 10.1016/j.clinbiochem.2004.05.019 15329318

[jat3814-bib-1003] Massart, L. M. , Vandenginste, B .G. M. , Buydens, L. M. C. , De Jong, S. , Lewi, P. J. , & Smeyers‐Verbeke, J. (1997). Handbook of Chemometrics and Qualimetrics: Part A, p. 200.

[jat3814-bib-0034] McElduff, A. , McElduff, P. , Wiley, V. , & Wilcken, B. (2005). Neonatal thyrotropin as measured in a congenital hypothyroidism screening program: influence of the mode of delivery. Journal of Clinical Endocrinology & Metabolism, 90(12), 6361–6363. 10.1210/jc.2005-0786 16144951

[jat3814-bib-0035] Milbrath, M. O. , Wenger, Y. , Chang, C. W. , Emond, C. , Garabrant, D. , Gillespie, B. W. , & Jolliet, O. (2009). Apparent half‐lives of dioxins, furans, and polychlorinated biphenyls as a function of age, body fat, smoking status, and breast‐feeding. Environmental Health Perspectives, 117(3), 417–425. 10.1289/ehp.11781 19337517 PMC2661912

[jat3814-bib-0036] Mocarelli, P. (2001). Seveso: A teaching story. Chemosphere, 43(4–7), 391–402. 10.1016/S0045-6535(00)00386-6 11372818

[jat3814-bib-0037] Mocarelli, P. , Gerthoux, P. M. , Patterson, D. G. Jr. , Milani, S. , Limonta, G. , Bertona, M. , … Needham, L. L. (2008). Dioxin exposure, from infancy through puberty, produces endocrine disruption and affects human semen quality. Environmental Health Perspectives, 116, 70–77. 10.1289/ehp.10399 18197302 PMC2199303

[jat3814-bib-0038] National Academies of Science, Engineering, and Medicine (2006). Health risks from dioxin and related compounds: Evaluation of the EPA reassessment. Washington, DC: National Academy Press Retrieved from http://www.nap.edu/catalog.php?record_id=11688

[jat3814-bib-0039] National Academies of Science, Engineering, and Medicine . (2011). Review of the Environmental Protection Agency's Draft IRIS Assessment of Formaldehyde. Access at http://dels.nas.edu/Report/Review‐Environmental‐Protection‐Agency/13142

[jat3814-bib-0040] National Academies of Science, Engineering, and Medicine (2014). Review of EPA's Integrated Risk Information System (IRIS) Process Washington, DC: National Academies Press 10.17226/18764 25101400

[jat3814-bib-0041] OHAT (2015a). OHAT risk of bias tool for human and animal studies. NC: Office of Health Assessment and Translation, Research Triangle Park.

[jat3814-bib-0042] OHAT (2015b). Handbook for conducting a literature‐based health assessment using OHAT approach for systematic review and evidence integration. National Institute of Environmental Health Sciences https://ntp.niehs.nih.gov/ntp/ohat/pubs/handbookjan2015_508.pdf

[jat3814-bib-0043] Parks, J. S. , Lin, M. , Grosse, S. D. , Hinton, C. F. , Drummond‐Borg, M. , Borgfeld, L. , & Sullivan, K. M. (2010). The impact of transient hypothyroidism on the increasing rate of congenital hypothyroidism in the United States. Pediatrics, 125(Suppl 2), S54–S63. 10.1542/peds.2009-1975F 20435718

[jat3814-bib-0044] Pesatori, A. C. (1995). Dioxin contamination in Seveso: the social tragedy and the scientific challenge. Giornale Italiano di Medicina del Lavoro Ed Ergonomia, 86(2), 111–124. 7659037

[jat3814-bib-0045] Pesatori, A. C. , Consonni, D. , Bachetti, S. , Zocchetti, C. , Bonzini, M. , Baccarelli, A. , & Bertazzi, P. A. (2003). Short‐ and long‐term morbidity and mortality in the population exposed to dioxin after the “Seveso accident”. Industrial Health, 41(3), 127–138. 10.2486/indhealth.41.127 12916742

[jat3814-bib-1002] Ranke, J. (2015). chemCal: Calibration Functions for Analytical Chemistry. R package version 0.1.37. https://CRAN.R‐project.org/package=chemCal

[jat3814-bib-1001] R Core Team . (2018). R: A language and environment for statistical computing. R Foundation for Statistical Computing, Vienna, Austria. Available online at https://www.R‐project.org/

[jat3814-bib-0047] Safe, S. H. (2000). Endocrine disruptors and human health—is there a problem? An update. Environmental Health Perspectives, 108(6), 487–493. 10856020 10.1289/ehp.00108487 PMC1638151

[jat3814-bib-0048] Saleh, D. S. , Lawrence, S. , Geraghty, M. T. , Gallego, P. H. , McAssey, K. , Wherrett, D. K. , & Chakraborty, P. (2016). Prediction of congenital hypothyroidism based on initial screening thyroid‐stimulating‐hormone. BMC Pediatrics, 16, 24 10.1186/s12887-016-0559-0 26839208 PMC4735969

[jat3814-bib-0049] SAC . (2010, 15 May 18). Technical comments on the derivation of cancer and non‐cancer toxicity criteria in EPA's reanalysis of key issues related to dioxin toxicity and response to NAS comments. Science Advisory Council. Retrieved from https://www.regulations.gov/document?D=EPA‐HQ‐ORD‐2010‐0395‐0051

[jat3814-bib-0051] Seo, B. W. , Li, M. H. , Hansen, L. G. , Moore, R. W. , Peterson, R. E. , & Schantz, S. L. (1995). Effects of gestational and lactational exposure to coplanar polychlorinated biphenyl (PCB) congeners or 2,3,7,8‐tetrachlorodibenzo‐p‐dioxin (TCDD) on thyroid hormone concentrations in weanling rats. Toxicological Letters, 78(3), 253–262. 10.1016/0378-4274(95)03329-J 7624895

[jat3814-bib-0052] Sewall, C. H. , Flagler, N. , Van den Heuvel, J. P. , Clark, G. C. , Tritscher, A. M. , Maronpot, R. M. , & Lucier, G. W. (1995). Alterations in thyroid function in female Sprague‐Dawley rats following chronic treatment with 2,3,7,8‐tetrachlorodibenzo‐p‐dioxin. Toxicology & Applied Pharmacology, 132(2), 237–244. 10.1006/taap.1995.1104 7540335

[jat3814-bib-0053] Signorini, S. , Gerthoux, P. M. , Dassi, C. , Cazzaniga, M. , Brambilla, P. , Vincoli, N. , & Mocarelli, P. (2000). Environmental exposure to dioxin: The Seveso experience. Andrologia, 32(4–5), 263–270. 10.1046/j.1439-0272.2000.00394.x 11021518

[jat3814-bib-0054] Simon, T. W. , Zhu, Y. , Dourson, M. L. , & Beck, N. B. (2016). Bayesian methods for uncertainty factor application for derivation of reference values. Regulatory Toxicology & Pharmacology, 80, 9–24. 10.1016/j.yrtph.2016.05.018 27211295

[jat3814-bib-0055] Urban, J. , Wikoff, D. , Haws, L. , Fitch, S. , Ring, C. , Thompson , & Suh, M. (2018). (2018, November 21). Systematic review and meta‐regression to characterize the dose‐response relationship between exposure to dioxin‐like compounds during sensitive windows of development and reduced sperm count. Zenodo. 10.5281/zenodo.1636357

[jat3814-bib-0056] USEPA . (2009). Summary of US EPA dioxin workshop. Cincinnati, Ohio.

[jat3814-bib-0057] USEPA . (2010a). EPA's reanalysis of key issues related to dioxin toxicity and response to NAS comments. (EPA/600/r‐10/038A). Washington, DC.

[jat3814-bib-0058] USEPA . (2010b). Recommended toxicity equivalence factors (TEFs) for human health risk assessments of 2,3,7,8‐tetrachlorodibenzo‐p‐dioxin and dioxin‐like compounds (EPA/100/R‐10/005) (p. 20460). Washington: DC.

[jat3814-bib-1000] USEPA . (2010c). SAB review of EPA's reanalysis of key issues related to dioxin toxicity and response to NAS comments (May 2010). (EPA‐SAB‐11‐014).

[jat3814-bib-0059] USEPA . (2012). EPA's reanalysis of key issues related to dioxin toxicity and response to NAS comments, Volume 1 (EPA/600/R‐10/038F). Washington, DC.

[jat3814-bib-0060] USEPA . (2018). Basic information about the Integrated Risk Information System. Retrieved December 1, 2017 https://www.epa.gov/iris/basic‐information‐about‐integrated‐risk‐information‐system

[jat3814-bib-0061] Van den Berg, M. , Birnbaum, L. S. , Denison, M. , De Vito, M. , Farland, W. , Feeley, M. , … Peterson, R. E. (2006). The 2005 World Health Organization reevaluation of human and mammalian toxic equivalency factors for dioxins and dioxin‐like compounds. Toxicological Science, 93(2), 223–241. 10.1093/toxsci/kfl055 PMC2290740 16829543

[jat3814-bib-0062] Van Landingham, C. , Mundt, K. A. , Allen, B. C. , & Gentry, P. R. (2016). The need for transparency and reproducibility in documenting values for regulatory decision making and evaluating causality: The example of formaldehyde. Regulatory & Toxicological Pharmacology, 81, 512–521. 10.1016/j.yrtph.2016.10.011 27771342

[jat3814-bib-0063] WHO . (1980). WHO laboratory manual for the examination of human semen and sperm‐cervical mucus interaction. Geneva, Switzerland: World Health Organization.

[jat3814-bib-0064] WHO . (1994). Indicators for assessing iodine deficiency disorders and their control through salt iodization. Geneva, Switzerland: World Health Organization.

[jat3814-bib-0065] WHO . (2010). WHO laboratory manual for HTE examination and processing of human semen: Fifth edition. Geneva, Switzerland: World Health Organization.

[jat3814-bib-0066] WHO . (2017). Guidance document on evaluating and expressing uncertainty in hazard characterization. Geneva, Switzerland: World Health Organization.

