# Supplementary material for: Application of qualitative and quantitative uncertainty assessment tools in developing ranges of plausible toxicity values for 2,3,7,8‐tetrachlorodibenzo‐p‐dioxin
Source: J Appl Toxicol. 2019 Jun 30;39(9):1293–310. doi: 10.1002/jat.3814 (PMC6771962; doi:10.1002/jat.3814)
Supplement: Supplementary file 1 — Data S1 Supporting information [file JAT-39-1293-s001.pdf]

Independent Assessment of Statistical Significance for Mocarelli et al. (2008) Quartile Analyses

Basing the analysis on the findings reported by Mocarelli *et al.* (2008) for the entire exposed group relative to the control group, and utilizing the general linear model implemented by the authors (which is equivalent to the standard two-sample t-test with homogenous variances), we evaluated significance as follows. Given a total sample size of 153 (82 in the control group and 71 in the exposed group), the degrees of freedom are equal to 151 (n_1+n_2-2), and the resulting t value is 2.26 for the comparison in sperm concentration between the control group and the entire exposed group. Assuming homogeneous variance across the control group and four quartile groups, assuming that each quartile consists of 18 samples (one group must actually have 17), and assuming that the mean of the first quartile group is no lower than the overall exposed group mean (an assumption supported by visual inspection of Figure 3 (panel A) in Mocarelli et al. (2008), the t statistic for a comparison of the control group and the first quartile group could be no higher than 1.41, which is non-significant with df=98. The reason for this difference is that the square root term would increase as a result of the smaller n in the exposed quartile (18) than in the entire exposed group (71). Thus, the sperm concentrations measured in the first quartile group are not significantly different from the control group.

An examination of the second and third quartiles revealed a similar result; neither the second nor third quartile is significantly different from the control group. Based on our independent assessment, the only statistically significant finding was that of the mean of the 4th quartile relative

to the control group. As such, statistically appropriate PODs from Mocarelli et al. (2008) would be 733 pt (median of the 4th quartile) or 210 ppt (the median of the entire exposed group).
