# Supplementary material for: Application of qualitative and quantitative uncertainty assessment tools in developing ranges of plausible toxicity values for 2,3,7,8‐tetrachlorodibenzo‐p‐dioxin
Source: J Appl Toxicol. 2019 Jun 30;39(9):1293–310. doi: 10.1002/jat.3814 (PMC6771962; doi:10.1002/jat.3814)
Supplement: Supplementary file 2 — Data S2 Supporting information [file JAT-39-1293-s002.pdf]

Bayesian Approach to Characterize Uncertainty Factors

A Bayesian approach to applying uncertainty factors was used, following the methods explained in NAS (2014). Effectively, this is an uncertainty propagation approach.

Under the traditional uncertainty-factor paradigm, a reference dose (RfD) would be derived by dividing the point of departure (POD) by one of a number standard uncertainty factors (UF).

$$\mathrm{RfD} = \mathrm{POD}/\mathrm{UF} \text{ (Equation 1)}$$

Here, the UF is represented differently by the factor U, which is expressed as 1/UF. This factor is intended to be protective of some "reasonably worst-case" scenario. For example, in the case of UF = 3 representing inter-individual variability (and thus U=1/3), the assumption is that sensitivity may vary among individuals, but setting the RfD at one-third of the POD should protect individuals up to some reasonable bound on sensitivity (e.g. perhaps 97.5% of the population).

The Bayesian approach applies a probabilistic framework to the traditional uncertainty-factor approach. The RfD is considered equal to the product of a POD and some factor U.

$$\mathrm{RfD} = \mathrm{POD} \times U \text{ (Equation 2)}$$

U is analogous to the factor of 1/UF in Equation 1: e.g., U = 1/3 to divide the POD by the standard UF of 3 representing inter-individual variability.

In the Bayesian approach, both the POD and U are considered probabilistically, as random variables obeying some known or estimated distributions. The RfD then also has a distribution, which can be characterized.[1]

The distribution of the POD is intended to represent uncertainty in the estimated POD, if known. In this analysis, a single distribution representing POD uncertainty was not derived. Rather, uncertainty was estimated qualitatively by comparing various PODs derived under various assumptions: e.g., various possible threshold levels where a specific neonatal TSH value (i.e., 5 uU/mL or 10 uU/mL) was linked to a specific exposure metric, which in this case would be maternal TCDD or maternal TEQ. Consequently, in this analysis, PODs were not represented by a distribution, but were treated as exact values. If a range of maternal TCDD and/or TEQ blood concentrations were available, the POD could be treated probabilistically.

The distribution of U is intended to represent the actual uncertainty or variability in the UF. For example, if U represents inter-individual variability, then the distribution should reflect the population distribution of sensitivity. If the POD is derived from human data, then the median individual would probably see adverse effects at a dose approximately equal to the POD (U = 1).

---

[1] In the language of Bayesian statistics, the distribution of the RfD is an "induced prior," rather than a posterior distribution (NAS, 2014). It reflects belief about what doses might be associated with a given adverse effect in the population, based only on what is known about the POD and what is believed to be true about the factor U. Bayesian inference methods could be used to update this induced prior with additional data from, for example, a new study about the exposure-response between maternal dioxin exposure and neonatal TSH levels in a large cohort with background-level exposures.

Some people will be less sensitive and would see adverse effects only at a dose much higher than the POD ($U > 1$). Some people will be more sensitive and would see adverse effects at a dose much lower than the POD ($U < 1$). If $U = 1/3$ is protective of, say, 97.5% of the population, then 97.5% of the distribution should be above 1/3.

Standard UFs are not linked to detailed data that could characterize an empirical distribution for U. Therefore, this analysis followed the approach used in NAS (2014) and Simon et al. (2016), assuming that the factor U obeys a log-normal distribution. The median of this distribution is set equal to 1, reflecting the assumption that the median individual would probably see adverse effects at a dose approximately equal to the POD ($U = 1$). To reflect the idea that a factor of 1/UF (where UF is some standard uncertainty factor) should be protective of 97.5% of the population, a log-scale standard deviation is chosen to ensure that the 2.5^{th} percentile of the distribution corresponds to 1/UF. For example, the distribution representing a standard uncertainty factor of 3 (for inter-individual variability) had a standard deviation chosen so that the 2.5^{th} percentile takes a value of 1/3: this is a log-scale standard deviation of 0.561. The distribution representing a standard uncertainty factor of 10 (for LOAEL-NOAEL extrapolation) had a log-scale standard deviation of 1.175. (NAS, 2014, provides a useful table of log-scale standard deviations corresponding to various standard UFs.)

Under the assumption that a POD is an exact value, and it is multiplied by an uncertainty factor U obeying a log-normal distribution (i.e., log U obeys a normal distribution with mean 0 and standard deviation equal to σ_U), then the resulting RfD obeys the following distribution on the log scale:

$$\log \text{RfD} \sim \text{Normal}(\mu = \log \text{POD}, \sigma = \sigma_U) \text{ (Equation 3)}$$

Under some circumstances, a traditional UF approach requires dividing the POD by two (or more) UFs. For example, if the POD is considered to be a LOAEL, then a UF of 10 is applied to represent uncertainty in converting from LOAEL to NOAEL, and then a UF of 3 is applied to represent inter-individual variability. In this Bayesian uncertainty-propagation framework, this situation can be represented as a POD successively multiplied by two uncertainty factors.

$$\text{RfD} = (\text{POD} \times U_1) \times U_2 \text{ (Equation 4)}$$

where log U_1 obeys a normal distribution with mean 0 and standard deviation σ_1, and log U_2 obeys a normal distribution with mean 0 and standard deviation σ_2. If U_1 and U_2 are uncorrelated, and if the POD is taken as an exact value (as before), then the resulting RfD obeys the following distribution on the log scale:

$$\log \text{RfD} \sim \text{Normal}\left(\mu = \log \text{POD}, \sigma = \sqrt{\sigma_1^2 + \sigma_2^2}\right) \text{ (Equation 5)}$$

The traditional UF example described above might apply a single combined UF of 30, because successively dividing by 10 and then by 3 is equivalent to simply dividing by 30. However, in the Bayesian framework, applying a single log-normal distribution representing a combined uncertainty factor U_1U_2 is *not* equivalent to successively applying two log-normal distributions representing U_1 and then U_2, as in Equation 5. For example, consider the case where U_1

represents the LOAEL-NOAEL conversion factor (2.5th percentile should be 1/10, so σ_l = 1.175), and U_2 represents inter-individual variability (2.5th percentile should be 1/3, so σ_2 = 0.561). A single combined UF of 30 would be represented by a distribution whose 2.5th percentile was 1/30, i.e. σ_U = 1.735. By contrast, applying the two uncertainty factors in succession (as in Equation 5) leads to a log-normal distribution, with $\sigma_U = \sqrt{\sigma_1^2 + \sigma_2^2} = 1.302$, and 2.5th percentile = 1/12.5 rather than 1/30. In fact, for this log-normal distribution (with σ_U = 1.302), 1/30 is the 1st percentile. This fact reflects the compounding of conservatism that occurs when a single combined UF is used. Because UFs can typically be assumed to represent uncorrelated sources of uncertainty or variability in the RfD, combining two UFs that are each intended to be conservative at a 97.5% level ultimately yields a single UF that is actually conservative at the 99% level.

Determination of POD values

The Bayesian approach was applied to derive distributions for RfDs corresponding to both maternal TCDD and maternal TEQ, associated with neonatal TSH threshold values of both 5 μU/mL and 10 μU/mL.

Because the only POD data provided by the USEPA was that for TCDD associated with a threshold value for neonatal TSH of 5uU/mL, PODs had to be determined for TCDD associated with a threshold of 10 uU/mL, as well as TEQ-based PODs associated with both neonatal TSH thresholds. To do so, the Bacarelli regression analyses were reproduced for both neonatal TSH

vs. maternal LASC TCDD, and neonatal TSH vs. maternal LASC TEQ. The data in Baccarelli Figure 2A (TCDD) and Figure 2D (TEQ) were digitized using Web Plot Digitizer software, and regression analysis (log neonatal TSH vs. log maternal LASC) was carried out using R (R Core Team, 2017). Using these regression models, inverse predictions were made of the maternal LASC TCDD and TEQ corresponding to neonatal TSH of 5 μU/mL and 10 μU/mL.

To derive PODs, the maternal LASC TCDD and TEQ values were converted into equivalent doses. For efficiency, rather than running the full Emond PBPK model, an *ad hoc* estimate of the relationship between dose and maternal LASC was developed by fitting a power curve to the maternal LASC (ppt) and equivalent point of departure (POD) doses (pg/kg-day) shown in EPA's sensitivity tree for Baccarelli (EPA Figure 4.7 in the dioxin reassessment). The best-fit model was

$$\text{POD} = \beta_0 + \beta_1 \text{LASC} + \beta_2 \text{LASC}^2 \text{ (Equation 6)}$$

where β_0 = -8.534e-1; β_1 = 5.433e-2; and β_2 = 1.441e-4.

The ad hoc estimated relationship was used to predict the doses corresponding to maternal LASC TCDD and TEQ corresponding to neonatal TSH of 5 μU/mL and 10 μU/mL. These equivalent doses were treated as PODs.

The Bayesian approach described above was then applied to each of these four PODs under two assumptions: first, that the POD represented a NOAEL (i.e., only applying a factor representing

inter-individual variability), and second, that the POD represented a LOAEL (i.e., successively applying two factors: one representing LOAEL-NOAEL extrapolation, and one representing inter-individual variability). The resulting RfD distributions are illustrated in Figure 6 in the main manuscript. To characterize the "reasonable worst-case scenario" from each of the RfD distributions, the 2.5th percentile was calculated for each distribution (representing an RfD that is conservative at the 97.5% level).

The results of each stage of this analysis are summarized in Supplemental Table S2-1: maternal LASC TCDD and TEQ values corresponding to neonatal TSH of 5 µU/mL and 10 µU/mL, the corresponding equivalent doses (PODs), and the corresponding 2.5th percentile RfD values.

Supplemental Table S2-1*

Neonatal TSH (uU/mL)	Maternal exposure metric	POD treated as	Maternal LASC (ppt)	POD (pg/kg-day)	RfD, 2.5th %ile (pg/kg-day)
5	TCDD	NOAEL	232.36	19.53	6.50
5	TEQ	NOAEL	489.94	60.30	20.08
10	TCDD	NOAEL	1506.15	407.72	135.78
10	TEQ	NOAEL	1755.72	538.57	179.36
5	TCDD	LOAEL	232.36	19.53	1.52
5	TEQ	LOAEL	489.94	60.30	4.70

10	TCDD	LOAEL	1506.15	407.72	31.77
10	TEQ	LOAEL	1755.72	538.57	41.97

* RfD lower-bound (2.5th percentile) values corresponding to various combinations of assumptions on threshold neonatal TSH, maternal exposure metric, and whether POD is treated as a NOAEL or LOAEL. Also provided: the maternal LASC corresponding to the given neonatal TSH value and maternal exposure metric; and the corresponding POD (i.e., the equivalent dose).
